# Efficient Construction of Atomic-Resolution Models of Non-Sulfated Chondroitin Glycosaminoglycan Using Molecular Dynamics Data

**DOI:** 10.3390/biom10040537

**Published:** 2020-04-02

**Authors:** Elizabeth K. Whitmore, Gabriel Vesenka, Hanna Sihler, Olgun Guvench

**Affiliations:** 1 Department of Pharmaceutical Sciences, University of New England College of Pharmacy, 716 Stevens Avenue, Portland, ME 04103, USA; ewhitmore@une.edu (E.K.W.); gvesenka1@une.edu (G.V.); hsihler@une.edu (H.S.); 2 Graduate School of Biomedical Science and Engineering, University of Maine, 5775 Stodder Hall, Orono, ME 04469, USA

**Keywords:** molecular dynamics, glycosaminoglycan, proteoglycan, chondroitin sulfate, carbohydrate conformation, carbohydrate flexibility, glycosidic linkage, ring pucker, force field, explicit solvent

## Abstract

Glycosaminoglycans (GAGs) are linear, structurally diverse, conformationally complex carbohydrate polymers that may contain up to 200 monosaccharides. These characteristics present a challenge for studying GAG conformational thermodynamics at atomic resolution using existing experimental methods. Molecular dynamics (MD) simulations can overcome this challenge but are only feasible for short GAG polymers. To address this problem, we developed an algorithm that applies all conformational parameters contributing to GAG backbone flexibility (i.e., bond lengths, bond angles, and dihedral angles) from unbiased all-atom explicit-solvent MD simulations of short GAG polymers to rapidly construct models of GAGs of arbitrary length. The algorithm was used to generate non-sulfated chondroitin 10- and 20-mer ensembles which were compared to MD-generated ensembles for internal validation. End-to-end distance distributions in constructed and MD-generated ensembles have minimal differences, suggesting that our algorithm produces conformational ensembles that mimic the backbone flexibility seen in simulation. Non-sulfated chondroitin 100- and 200-mer ensembles were constructed within a day, demonstrating the efficiency of the algorithm and reduction in time and computational cost compared to simulation.

## 1. Introduction

The diverse group of protein–carbohydrate conjugates called proteoglycans (PGs) is a fundamental component of tissue structure in animals and can be found in the extracellular matrix (ECM) as well as on and within cells. PGs bind growth factors [1,2,3,4,5,6,7,8,9,10,11,12], enzymes [2,12], membrane receptors [12], and ECM molecules [2,12,13]. By doing so, they modulate signal transduction [13,14], tissue morphogenesis [2,8,9,10,11], and matrix assembly [2,15,16,17]. PG bioactivity is often dependent on the covalently linked carbohydrate chains called glycosaminoglycans (GAGs), which are linear, highly negatively charged, and structurally diverse carbohydrate polymers. GAGs mediate receptor–ligand complex formation by either forming non-covalent complexes with proteins or inhibiting the formation of complexes with other biomolecules. This makes GAGs key modulators in many diseases, giving them potential therapeutic applications. For example, heparan sulfate (HS) is released during sepsis and induces septic shock [18,19]; the removal of chondroitin sulfate (CS) may enhance memory retention and slow neurodegeneration in patients with Alzheimer’s disease [20,21,22]; and dermatan sulfate (DS) deficiency has been implicated in Ehlers–Danlos syndrome, thus the screening of DS in urine could be used as an early diagnostic tool [23,24].

**Figure 1:**
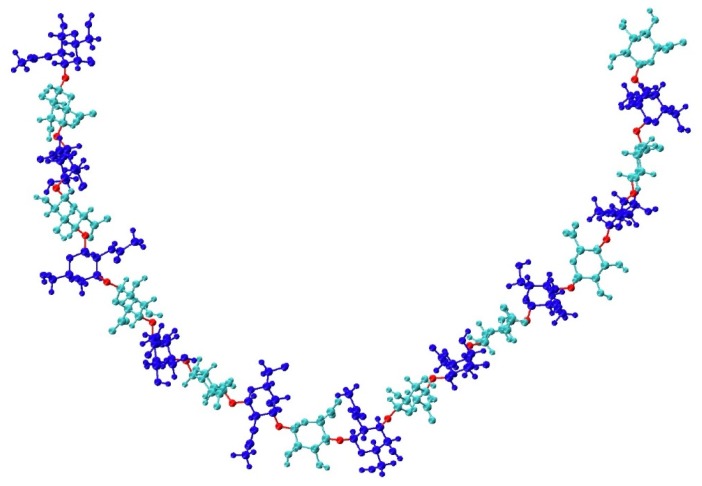
Compact non-sulfated chondroitin 20-mer conformation arising from flexible glycosidic linkages (red) between monosaccharide rings (GalNAc in blue and GlcA in cyan). The molecular graphics throughout are produced with the VMD program [57].

GAG binding sites on proteins are determined by protein sequence and structure, with requirements for both shape and charge complementarity [12,25]. Thus, GAG function depends on GAG three-dimensional structure and conformation. Even subtle structural differences impact GAG function. For example, while CS and DS have many functional differences, the only structural difference is in the chirality of the uronic acid monosaccharides. While much is known about GAG function, attempting to study GAG conformational thermodynamics at atomic resolution presents a largely unsolved problem for existing experimental methods. This is largely due to the structural and conformational complexities of GAGs. For example, a given GAG consists of a repeating sequence of a particular disaccharide, but conformational complexity is introduced through flexibility in the glycosidic linkages between monosaccharides [26,27,28,29,30] (Figure 1). Additional complexity results from non-template-based synthesis [31] and variable enzymatic sulfation [32], which means a biological sample of a GAG composed of a specific disaccharide repeat will be polydisperse and heterogeneous owing to the variable length and sulfation of the individual polymer molecules. Liquid chromatography–mass spectrometry (LC-MS) [33,34,35], X-ray crystallography [36,37,38,39,40,41], and nuclear magnetic resonance (NMR) [42,43,44,45] are used to study GAGs but are limited in their ability to account for all of these complexities. Additionally, some studies have used results from LC-MS [45], X-ray crystallography [46], and NMR [46,47,48,49,50,51] to compare and validate conformational data from molecular dynamics (MD) simulations. This suggests that MD simulations can produce results complementary to experimental analysis methods by providing realistic three-dimensional atomic-resolution molecular models of GAG conformational ensembles [52,53,54,55,56].

A critical challenge with MD simulations of GAGs is that a single biological GAG polymer chain may contain up to 200 monosaccharide units [9]. When fully solvated, the resulting system will have in excess of 10^6^ atoms. It is not feasible to routinely simulate such a system using current graphics processing unit (GPU)-accelerated MD codes with a modern GPU and multi-core CPU. This limits the utility of all-atom explicit-solvent MD as a tool for routine conformational analysis of GAGs of this size.

Coarse-grained (CG) MD simulations are the most feasible current alternative to all-atom explicit-solvent MD as they entail fewer degrees of freedom for the solute [48] and often an implicit (continuum) description of the solvent [58,59]. This can make CG MD two to three orders of magnitude faster, thereby allowing for the handling of large systems [60], such as GAG 200-mers. Indeed, a recent CG model using glycosidic linkage and ring pucker energy functions has provided previously-unseen details of the structure–dynamics relationship of GAGs in the context of PGs [48]. An important insight from that study was that GAGs, in contrast to the unique ordered conformations of folded proteins, need to be considered as existing in conformational ensembles containing a large diversity of three-dimensional conformations.

As an alternative approach to using CG MD to generate such conformational ensembles for GAGs, we propose using glycosidic linkage and monosaccharide ring conformations from unbiased all-atom explicit-solvent MD simulations [56,61,62,63] of short GAG polymers to rapidly construct conformational ensembles for GAGs of an arbitrary length. Toward this end, we studied a non-sulfated chondroitin 20-mer with the sequence [-4 glucuronate β1-3 N-acetylgalactosamine β1-]_10_ for its simplicity and homogeneity. We first ran microsecond-scale all-atom explicit solvent MD on the 20-mer and used the resulting trajectories to develop a database of conformations. From this database, we randomly selected individual values for the bond lengths, bond angles, and dihedral angles in the glycosidic linkages connecting glucuronate (GlcA) and N-acetylgalactosamine (GalNAc) and in the monosaccharide rings. These values were used to construct a 20-mer conformational ensemble. The comparison of the constructed ensemble with the MD-generated ensemble of 20-mer conformations revealed similar end-to-end distance distributions, with a strong bias toward extended conformations in both cases. Short end-to-end distances associated with more compact conformations were facilitated by the sampling of non-^4^C_1_ ring puckering by GlcA. This change in ring geometry, which occurs rarely on the microsecond timescale, introduced kinks into the polymer, causing it to bend back toward itself. The fact that the MD-generated ensemble had a great deal of variability in both end-to-end distances and radii of gyration demonstrates the inherent flexibility of the chondroitin polymer in aqueous solution. The fact that the constructed ensemble has very similar conformational properties to the MD-generated ensemble suggests that there is little correlation between the individual dihedral angle values that determine the internal geometry of a given conformation. Therefore, on the timescale of the simulations, non-sulfated chondroitin 20-mer does not appear to have any higher-order structure, in contrast to, for example, the secondary and tertiary structure seen in proteins. This lack of higher-order structure was borne out in a comparison of end-to-end distances for constructed vs. MD-generated ensembles of 10-mers, with the constructed ensemble built using the 20-mer database. Finally, we used the methodology to produce conformational ensembles of 100-mers and of 200-mers. The ability to model polymers with biologically-relevant chain lengths (e.g., 100- to 200-mers) will provide insights into GAG binding by other biomolecules. This will be especially useful in understanding the formation of complexes containing multiple biomolecules bound to a single GAG.

Other programs that construct three-dimensional atomic-resolution models of GAG polymers exist, for example, Glycam GAG Builder [64], POLYS Glycan Builder [65], CarbBuilder [66], and MatrixDB GAG Builder [67,68], which allow the user to choose GAG type, length, and sequence and are useful tools for producing an initial structure for MD simulations. Glycam and POLYS Glycan Builder allow the user either to specify particular glycosidic linkage dihedral angle values or use default parameters pulled from their databases. The databases used by Glycam, POLYS Glycan Builder, and Carb Builder include GAG mono- and disaccharide structures determined by molecular mechanics and/or MD. MatrixDB pulls from databases of experimentally determined conformations of GAG disaccharides from crystallized GAG–protein complexes. While the user has the option to choose the GAG length, these tools are intended for shorter GAG polymers. In contrast to these tools, our algorithm pulls from a database of full conformational landscapes of unbound GAG 20-mers. Additionally, our algorithm is intended for modeling long GAG polymers with biologically-relevant chain lengths and can quickly produce large ensembles (e.g., on the order of 10,000 3-D models) of polymer conformations that we would expect to see in simulation. Thus, it eliminates the need for simulation, reducing time and computational cost.

## 2. Materials and Methods

### 2.1. Molecular Dynamics

#### 2.1.1. System Construction

Coordinates for all systems were constructed using the CHARMM software [69,70,71] v. c41b2 with the CHARMM36 (C36) biomolecular force field for carbohydrates [56,61,62,63]. Of note, it has been shown that MD simulations can reproduce ring puckers observed by NMR [49,50,51], with one study demonstrating the capacity of the CHARMM36 force field to reproduce NMR data for an iduronate derivative in the context of a heparin analogue [51]. The initial conformation for an MD simulation of non-sulfated chondroitin 20-mer was fully extended, with glycosidic linkage dihedrals *ϕ* = −83.75° and *ψ* = −156.25° in all GlcAβ1-3GalNAc linkages and *ϕ* = −63.75° and *ψ* = 118.75° in all GalNAcβ1-4GlcA linkages. These glycosidic linkage dihedral angle values were found to be the most energetically favorable in MD-simulated, non-sulfated chondroitin disaccharides [54]. All other internal coordinates were taken from the force-field files. In this conformation, the 20-mer had an end-to-end distance of 101.8 Å and was solvated in a cubic periodic unit cell with an edge length of 124.3 Å (~63,000 water molecules). The explicit solvent consisted of the TIP3P water model [72,73], neutralizing Na+ counterions, and 140 mM sodium chloride.

#### 2.1.2. Energy Minimization and Heating

The NAMD program [74] v. 2.12 (http://www.ks.uiuc.edu/Research/namd/) was used to minimize the potential energy for 1000 steps using the conjugate gradient method [75,76] then heat the system to the target temperature of 310 K by reassigning velocities from a random distribution at the target temperature every 1000 steps for 20,000 steps with a timestep of 0.002 ps (40 ps). During heating, harmonic positional restraints were placed on non-hydrogen atoms of the solute and constraints [77,78,79] were applied to maintain equilibrium values for TIP3P geometries and for bond lengths involving hydrogen atoms. The Lennard–Jones (L-J) [80] and electrostatic potential energies had cutoff distances of 10 Å. An energy switching function [81] was applied to L-J interactions between 8 and 10 Å and an isotropic pressure correction accounted for contributions from L-J interactions beyond the cutoff [82]. The particle mesh Ewald (PME) method [83] with fourth order B-spline interpolation for a cubic unit cell and fast Fourier transform (FFT) grid spacing of 1.0 Å along each axis was used to account for electrostatic interactions beyond the cutoff. Consistent with the CHARMM additive force fields for proteins, nucleic acids, lipids, and small molecules [84], carbohydrate 1-4 non-bonded interactions were not scaled (i.e., scaling factor = 1.0). Heating was done under constant pressure with pressure regulated at 1 atm by a Langevin Piston barostat [85]. A 500,000-step (1-ns) unbiased constant particle number/constant pressure/constant temperature (NPT) MD run, followed with a temperature of 310 K maintained by a Langevin thermostat [86] and without positional restraints. The average periodic cell parameters from the last half of this NPT ensemble trajectory (123.7 Å) were used as cell basis vectors for the quadruplicate canonical (NVT) ensemble MD simulations detailed below. These were preceded by minimization and heating as detailed above, with the exception of constant volume (i.e., no Langevin piston barostat) with a box edge length of 123.7 Å.

#### 2.1.3. Production Simulations

Unbiased canonical (NVT) ensemble MD was run using CHARMM software with the OpenMM GPU acceleration interface [87,88,89,90,91,92] on CUDA platform and GTX 1080 Ti graphics cards (NVIDIA Corp., Santa Clara, USA.). Non-bonded interaction truncations and energy calculations were performed using the same methods from the heating stage, and Ewald summation of Gaussian electrostatic charge density distributions [93,94] with a width of 0.320 was performed. The SHAKE algorithm [77] was used to constrain all water geometries and bonds involving hydrogen atoms using bond distances from the parameter table and the leapfrog Verlet integration algorithm [95] was used for Langevin dynamics with a friction coefficient of 0.1 ps^−1^, a constant temperature of 310 K, and a 0.002-ps timestep. Prior to production, each of four replicates was equilibrated for 50,000 steps (100 ps). Simulations were run for 250,000,000 steps (500 ns) and atomic coordinates were saved at 25,000-step (50-ps) intervals for analyses (10,000 snapshots per quadruplicate simulation).

#### 2.1.4. Conformational Analysis

**Figure 2:**
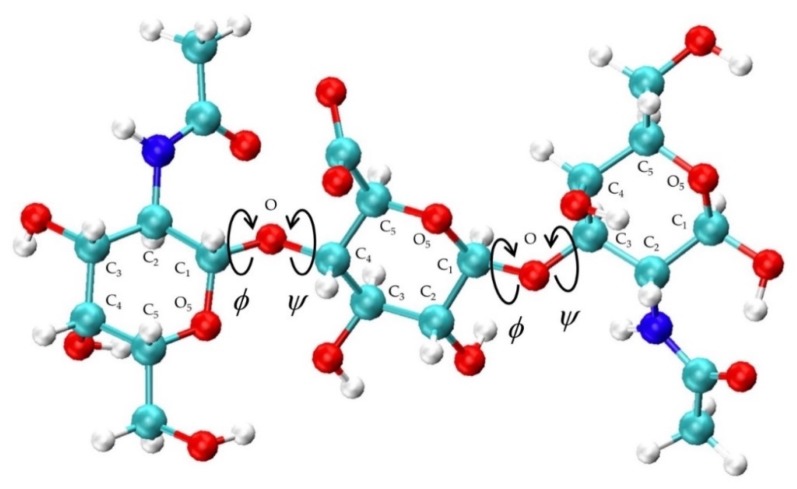
Non-sulfated chondroitin trisaccharide with glycosidic linkage dihedral angles, monosaccharide ring atoms, and linker oxygen atoms labeled; glycosidic linkage parameters used in the construction algorithm include C_1_-O and O-C*_n_* bond lengths, O_5_-C_1_-O and C_1_-O-C*_n_* bond angles, and *ϕ* = O_5_-C_1_-O-C*_n_* and *ψ* = C_1_-O-C*_n_*-C_(*n*−1)_ dihedral angles.

Chondroitin conformational properties were quantified by bond length, bond angle, and dihedral angle values. Glycosidic linkage conformational values considered were bond lengths for C_1_-O and O-C*_n_*, bond angles defined by O_5_-C_1_-O and C_1_-O-C*_n_*, and *ϕ* and *ψ* dihedral angles with IUPAC definitions: *ϕ* = O_5_-C_1_-O-C*_n_* and *ψ* = C_1_-O-C*_n_*-C_(*n*−1)_ (Figure 2). Glycosidic linkage dihedral free energies Δ*G*(*ϕ*, *ψ*) were analyzed to characterize potential conformational patterns. *ϕ*, *ψ* dihedral values from the MD-generated 20-mer ensemble are taken to have uniform probabilities. Δ*G*(*ϕ*, *ψ*) is therefore computed by binning these values into 2.5° × 2.5° bins and then using the relationship Δ*G*(*ϕ_i_*,*ψ_j_*) = -*RT*ln(*n_ij_*) – *k*, where *n_ij_* is the bin count for the bin corresponding to *ϕ_i_*, *ψ_j_*, *R* is the universal gas constant, *T* is the temperature of the MD simulations, and *k* is chosen so that the global minimum is located at Δ*G* = 0 kcal/mol.

Geometric values defining monosaccharide ring conformations included all bond lengths, bond angles, and dihedral angles within the ring and in exocyclic functional groups that are not part of a glycosidic linkage. To characterize potential conformational patterns in monosaccharide rings, Cremer-Pople (C-P) ring-puckering parameters (*ϕ*, *θ*, *Q*) of each monosaccharide ring in the MD-simulated 20-mer ensemble were computed. Conformations of each element (i.e., each GlcA monosaccharide, GalNAc monosaccharide, β1-3 linkage, and β1-4 linkage) were extracted separately from each saved snapshot of the 20-mer MD trajectories. Initially, data were separated out by run and residue/linkage number and aggregated across all snapshots in each run to determine if conformational data were the same in different runs and if individual linkage and ring conformations are dependent upon one another. Subsequently, all individual conformations were aggregated across all snapshots in all runs (e.g., 10,000 snapshots * 4 runs * 10 GlcA monosaccharides = 400,000 samples of GlcA monosaccharide conformations) to yield one set of data for each of: (1) GlcA monosaccharide conformation, (2) GalNAc monosaccharide conformation, (3) β1-3 linkage conformation, and (4) β1-4 linkage conformation.

### 2.2. Construction Algorithm to Generate GAG Conformational Ensembles

The conformational data described above served as inputs to an algorithm we developed to generate chondroitin polymer conformational ensembles of user-specified length and with a user-specified number of conformations. The algorithm works as follows:

1. In each constructed polymer conformation, each glycosidic linkage and monosaccharide ring is treated independently, and conformational parameters are randomly selected from the database containing the corresponding linkage or ring conformations from the 20-mer MD trajectories;
2. Two CHARMM stream files are written, one to define the sequence and linkages in the polymer and another to perform the following procedure for each frame: (1) All internal geometry conformation values selected by the algorithm are assigned and used to construct atomic coordinates. (2) End-to-end distance (i.e., distance between C_1_ of the reducing end and C_4_ of the non-reducing end) and radius of gyration are calculated. (3) A 100-step steepest descent (SD) potential energy minimization followed by a 100-step conjugate gradient minimization, each with intramolecular restraints, is performed to relieve bonded strain and steric clashes. The Lennard–Jones potential ($E_{L-J}$) is calculated on an atom–atom pair (i,j) basis using an energy switching function, as implemented in CHARMM with *r*_on_ = 7.5 Å and *r*_off_ = 8.5 Å [69]. As there is no solvent and thus no solvent screening of electrostatic interactions, electrostatics are excluded from energy calculations to prevent the non-physical intramolecular association of charged and polar groups. All glycosidic linkage and endocyclic ring dihedral angles, along with a dihedral angle in each GlcA carboxylate group (C_4_-C_5_-C_6_-O_61_) and GalNAc N-acetyl group (C-N-C_2_-C_3_), are restrained to their starting values (i.e., those randomly selected from the database) during minimization so as not to change the conformations observed in simulation. Dihedral restraint energy ($E_{\mathrm{rdihe}}$) is calculated by comparing each restrained dihedral angle’s database value ($\phi_{0}$) to its value ($\phi_{1}$) in the current frame of minimization with a force constant ($k_{\mathrm{dihe}}$) of 100.0 kcal/mol/radian/radian (Equation (1))
  (1) $$E_{\mathrm{rdihe}}=k_{\mathrm{dihe}}*\sum {\left(\phi_{1}-\phi_{0}\right)}^{2}$$
3. To ensure conformational ensembles do not contain non-physical conformations, a bond potential energy ($E_{b}$) cutoff is applied. This cutoff is the sum of a polymer-length-specific cutoff and a constant independent of polymer length. The length-specific component of the cutoff is the bond potential energy after energy minimization, performed using the same restraints and minimization protocol used for each frame of the constructed ensemble (outlined above), of the polymer constructed in a fully-extended conformation (i.e., with the same glycosidic linkage *ϕ* and *ψ* angles as the starting conformation for MD simulations). The constant is added as a buffer to account for slight variations in the energies of other extended conformations. As linkage and ring conformations are treated independently and selected at random, it is possible to have a bond piercing another monosaccharide ring that may not be corrected by minimization. To estimate the ring-piercing bond strain energy for each exocyclic bond not participating in a glycosidic linkage, a system containing two non-bonded monosaccharides (i.e., GlcA and GalNAc, GlcA and GlcA, or GalNAc and GalNAc) was constructed such that an exocyclic bond of one monosaccharide pierces the ring of the other. To estimate the bond strain energy for each bond participating in a glycosidic linkage, a system containing one disaccharide unit (i.e., GlcAβ1-3GalNAc or GalNAcβ1-4GlcA) and a single monosaccharide (i.e., GlcA or GalNAc) was constructed such that a linkage bond in the disaccharide pierces the ring of the single monosaccharide. Systems containing interlocking rings (i.e., GlcA-GalNAc, GlcA-GlcA, and GalNAc-GalNAc) were also constructed to estimate the bond strain energy of the bonds piercing the opposite ring. The same energy minimization protocol used in the algorithm was performed on this conformation, as well as a conformation in which the non-bonded saccharide units are 20 Å apart, and the post-minimization lengths of the bond piercing the ring in the initial conformation were compared. The pierced bond length ($x_{2}$), the non-pierced bond length ($x_{1}$), and the equilibrium bond length ($x_{0}$) and corresponding force-field bond-stretching constant ($k_{b}$) from the CHARMM parameter file were used to estimate a lower bound on the energy ($\Delta E_{b}$) resulting from the bond distortion (Equation (2)).
  (2) $$\Delta E_{b}=k_{b}*\left[{\left(x_{2}-x_{0}\right)}^{2}-{\left(x_{1}-x_{0}\right)}^{2}\right]$$
  Of all conformations that still had a bond piercing a ring after minimization, the smallest $\Delta E_{b}$ = 132.3 kcal/mol. Of the conformations in which ring piercing was corrected during minimization, the maximum $\Delta E_{b}$ < 1 kcal/mol. Thus, a buffer of 100 kcal/mol is added to the post-minimization bond potential of the initial extended conformation for any given polymer length. If the post-minimization bond potential of a given frame is beyond this cutoff, the frame is excluded from the ensemble.

For internal validation of our implementation of the algorithm, bond length probability distributions for each type of bond (i.e., C-C single bond, C-O single bond, C=O double bond, C-O partial double bond of GlcA carboxylate group, C_2_-N single bond between GalNAc amide and ring carbon, C-N single bond within GalNAc amide, C-H bond, O-H bond, and N-H bond), free energies Δ*G*(*ϕ*, *ψ*) for β1-3 and β1-4 glycosidic linkages, C-P parameters of GlcA and GalNAc monosaccharide rings, end-to-end distance distributions, and scatterplots of radius of gyration as a function of end-to-end distance from MD-generated ensembles and constructed ensembles both before and after energy minimization were compared. Additionally, bond potential energy distributions from constructed ensembles after energy minimization were plotted to verify that the algorithm calculated an appropriate energy cutoff and gave the expected energy distributions for the given polymer size.

To assess the expediency of application of MD-generated 20-mer conformations to construct chondroitin polymers of variable length, we constructed a non-sulfated chondroitin 10-mer ensemble using the algorithm and compared it to chondroitin 10-mer conformational ensembles generated by MD using the same protocol as the 20-mer simulations. We also constructed conformational ensembles of a non-sulfated chondroitin 100-mer and 200-mer to demonstrate the efficacy and efficiency of our algorithm to construct conformational ensembles of chondroitin polymers with biologically-relevant chain lengths.

## 3. Results and Discussion

### 3.1. Glycosidic Linkage Geometries

**Figure 3:**
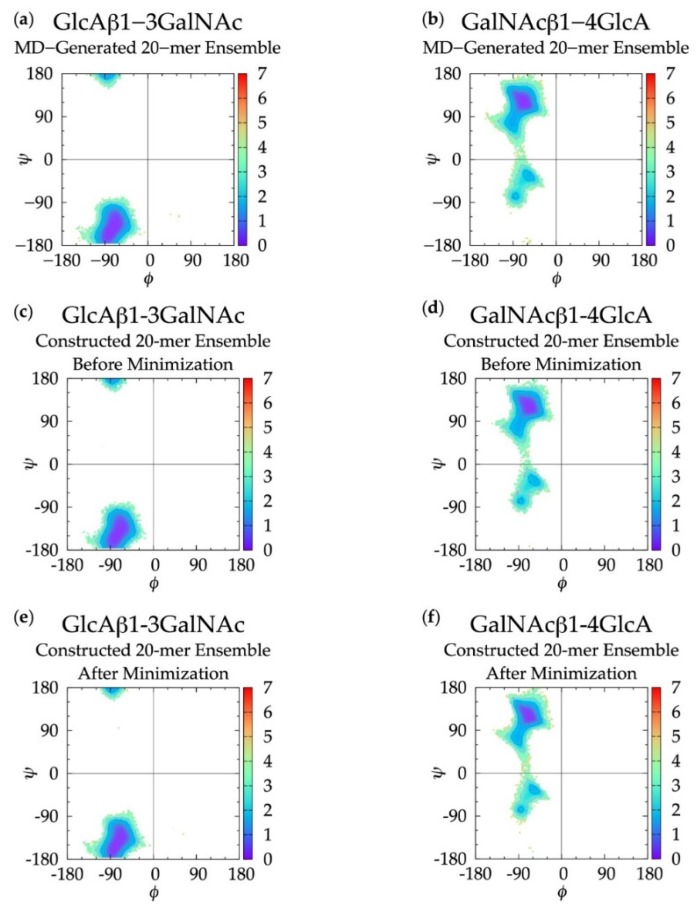
∆*G*(*ϕ*, *ψ*) in non-sulfated chondroitin 20-mer ensembles for aggregated GlcAβ1-3GalNAc and GalNAcβ1-4GlcA glycosidic linkage data (**a**,**b**) MD-generated ensembles, (**c**,**d**) constructed ensembles before minimization, and (**e**,**f**) constructed ensembles after minimization; contour lines every 1 kcal/mol.

In non-sulfated chondroitin 20-mer MD simulations, we found that all *ϕ*, *ψ* dihedrals sampled in GlcAβ1-3GalNAc linkages were centered about a global free energy minimum (Min I) while GalNAcβ1-4GlcA linkages showed more flexibility. In addition to a global minimum, Δ*G*(*ϕ*, *ψ*) for GalNAcβ1-4GlcA also has two local minima (Min II and Min II’) (Figure 3 and Table 1). To validate these observed glycosidic linkage geometries, we looked at the free energy minima of non-sulfated chondroitin glycosidic linkage dihedrals from biased MD simulations of disaccharides (using dihedral definitions *ϕ* = O_5_-C_1_-O-C*_n_* and *ψ* = C_1_-O-C*_n_*-C_(*n+*1)_ as opposed to the IUPAC *ψ* = C_1_-O-C*_n_*-C_(*n*−1)_ used in our study) [54] (Table S1). We found that at each free energy minimum in β1-3 and β1-4 linkages, our *ϕ* dihedrals differed by no more than +/-2.5° and our *ψ* dihedrals differed by no more than +/-127.5°, which is in close agreement if we assume C_1_-O-C_3_-C_2_ = C_1_-O-C_3_-C_4_ + 120° and C_1_-O-C_4_-C_3_ = C_1_-O-C_4_-C_5_ - 120°. Additionally, our data were mostly in agreement with the most energetically-favorable glycosidic linkage dihedrals (i.e., at global minima) in non-sulfated chondroitin hexasaccharides from MD simulations (using dihedral definitions *ϕ* = O_5_-C_1_-O-C*_n_* and *ψ* = C_1_-O-C*_n_*-C_(*n+*1)_) and validated by NMR [96] (Table S1). The biggest difference was in our β1-3 *ψ* dihedrals, which differed by about +100° (+120° difference expected). This study restrained pyranose rings to a ^4^C_1_ chair and did not use explicit solvent in simulations. Each of these factors may contribute to interactions between neighboring monosaccharides and thus glycosidic linkage conformation, which would explain the variation from our results.

For internal validation of the construction algorithm, we compared glycosidic linkage input and output data. If the algorithm is performing correctly, Δ*G*(*ϕ*, *ψ*) from the MD ensemble and the constructed ensemble *before minimization* will be nearly identical, and Δ*G*(*ϕ*, *ψ*) from the constructed ensemble *after minimization* will not be substantially different. Performing the comparison between the ensemble of 40,000 20-mer conformations from the MD and a constructed ensemble of the same size confirms this to be the case. Figure 3 demonstrates that Δ*G*(*ϕ*, *ψ*) for GlcAβ1-3GalNAc and for GalNAcβ1-4GlcA glycosidic linkages are qualitatively identical when comparing the MD-generated and constructed ensembles. Quantitative analysis (Table 1) shows that the global minima (Min I) for both types of linkages and the secondary local minima (Min II and Min II’) for GalNAcβ1-4GlcA linkages are basically identical with 0° to 5° differences between the MD-generated input data and the constructed ensemble output data before minimization. The minimization in the construction algorithm, used to resolve any steric clashes, results in relatively minor changes in the location of the global minima, also ranging from 0° to 5°. Constructed ensemble glycosidic dihedral values after minimization are within 5° of the MD data, and Δ*G*(*ϕ*, *ψ*) values are within 0.1 kcal/mol.

As detailed in Methods and discussed below, the minimization portion of the construction algorithm not only relieves any steric clashes, but also is used to detect bond-strain energies indicative of ring piercing. The close similarity of Δ*G*(*ϕ*, *ψ*) for the constructed ensemble after minimization in comparison to the MD-generated ensemble suggests that few constructed conformations have large steric clashes, resulting in large conformational shifts after minimization. It also suggests that a few constructed conformations have ring-piercing events that necessitate their exclusion from the constructed ensemble altogether. Indeed, this is the case: during the creation of the 40,000-member constructed ensemble, only 18 conformations were excluded because they failed to meet the bond-strain energy criterion.

### 3.2. GlcA Ring Pucker Effects

**Figure 4:**
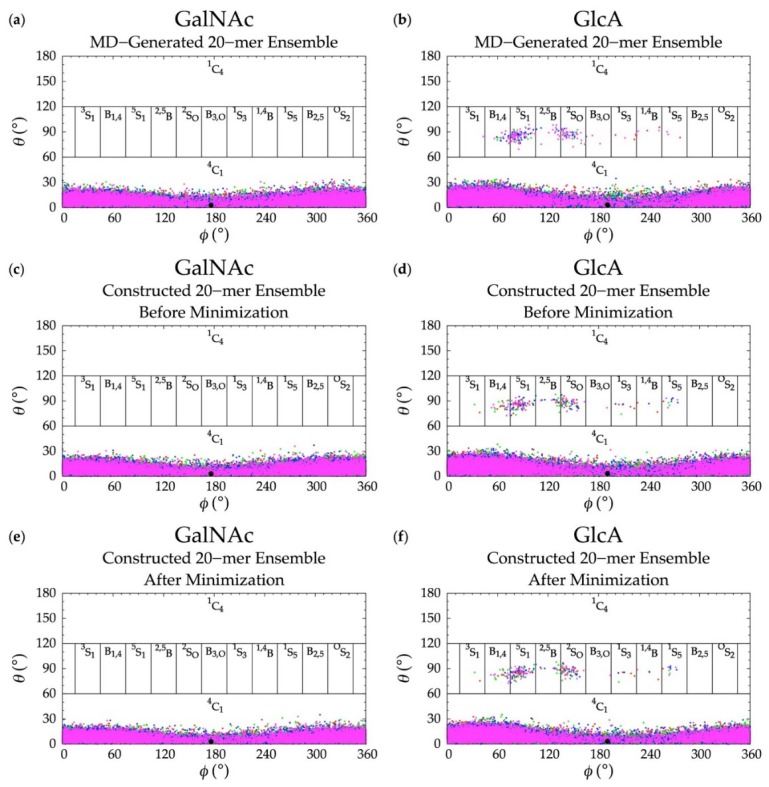
Cremer–Pople data for GalNAc and GlcA in (**a**,**b**) MD-generated ensembles and constructed ensembles (**c**,**d**) before and (**e**,**f**) after energy minimization; geometries from the four sets of each type of ensemble are represented by red, green, blue, and magenta dots, respectively and the force-field geometry is represented by a single large black dot. Cremer–Pople parameters (*ϕ*, *θ*) for all rings in every tenth snapshot from each ensemble were plotted (i.e., 10 rings * 1,000 snapshots per run * 4 runs = 40,000 parameter sets). As the algorithm reads all ring conformations sampled in MD, not all datapoints in panels (**c**–**f**) are seen in panels (**a**,**b**) but the full MD-generated dataset contains all datapoints in the constructed ensembles.

An initial implementation of the construction algorithm used default force-field geometries for all GalNAc and GlcA rings. The result was that all GalNAc and GlcA rings in an algorithmically-constructed conformation had the same internal geometries. Ensembles constructed using this version of the algorithm had longer average end-to-end distances than MD-generated ensembles (Figure S1), which meant that, on average, constructed conformations were overly extended. The default force-field ring pucker geometry for both types of monosaccharides was ^4^C_1_. With that ring pucker, all βGlcA and all but one βGalNAc exocyclic functional groups are equatorial, and therefore the ^4^C_1_ ring pucker is expected to be strongly preferred to other ring pucker geometries. To validate this simple approach to assigning ring pucker geometry, we computed C-P parameters of each monosaccharide ring in the MD-simulated 20-mer ensemble (10 * 40,000 = 400,000 ring conformations for each of the two monosaccharide types). As seen in NMR and force-field studies, the stable ^4^C_1_ chair ring pucker was the principal conformer for both GlcA [50,96,97,98,99] and GalNAc [46,96,98,100] in the MD simulations, with slight deformations (0° < C-P *θ* < 30°) (Figure 4a,b). However, a small minority of GlcA ring conformers were skew-boat or boat, namely ^3^S_1_, B_1,4_, ^5^S_1_, ^2,5^B, ^2^S_O_, B_3,O_, ^1^S_3_, ^1,4^B, and ^1^S_5_ (60° < C-P *θ* < 120°) (Figure 4b). Studies that performed unbiased MD simulations with other force fields observed skew-boat and boat ring puckers of non-sulfated GlcA monosaccharides on the microsecond timescale, but the occurrences were negligible due to high energy barriers [50,98]. In line with those findings, we observed only occasional GlcA skew-boat and boat pucker transitions in chondroitin 20-mers in our 500-ns unbiased CHARMM simulations. However, the C-P *ϕ* values in non-^4^C_1_ GlcA conformers in these studies differed from ours. Specifically, one study found ^2^S_O_, B_3,O_, ^1^S_3_, ^1,4^B, and ^1^S_5_ [98]. Slight differences could be explained by differing ion concentrations which likely impacted pyranose ring puckers [101]. However, it is likely that the differences primarily result from intramolecular interactions. The aforementioned literature data come from simulated GlcA monosaccharides only, whereas our results come from simulated chondroitin 20-mers.

**Figure 5:**
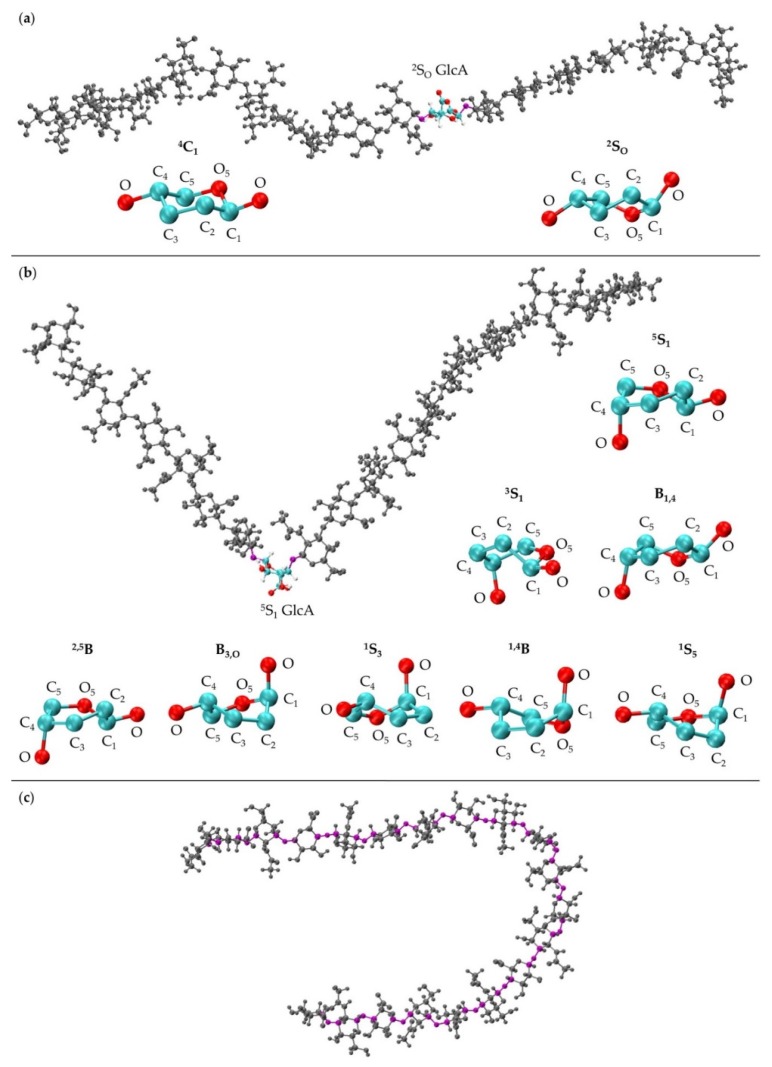
(**a**) 20-mer conformation with a ^2^S_O_ GlcA conformer (colored by atom type with flanking linkage atoms highlighted in purple) and close-ups of GlcA monosaccharide rings in ^4^C_1_ and ^2^S_O_ conformations (shows endocyclic ring atoms and linker oxygen atoms only); (**b**) 20-mer conformation with a kink at a ^5^S_1_ GlcA conformer (colored by atom type with flanking linkage atoms highlighted in purple) and GlcA monosaccharide rings in ^5^S_1_, ^3^S_1_, B_1,4_, ^2,5^B, B_3,O_, ^1^S_3_, ^1,4^B, and ^1^S_5_ conformations (shows endocyclic ring atoms and linker oxygen atoms only); (**c**) 20-mer conformation with a curve caused by flexible glycosidic linkage geometries (highlighted in purple) and all monosaccharides in ^4^C_1_ conformations; all images came from MD-generated ensembles.

The MD-generated 20-mer GlcA ring conformations can be separated into two broad categories: those that do not introduce a kink into the polymer and those that do. With the inclusion of ^4^C_1_, the former category encompasses ^4^C_1_ and ^2^S_O_ GlcA ring puckers, both of which place the two glycosidic linkage oxygen atoms, located at opposite ends of the ring, in an equatorial conformation (Figure 5a). As such, the O-C_1_ and C_4_-O bond vectors therein are approximately parallel and promote extended polymer conformations. The latter category encompasses ^3^S_1_, B_1,4_, ^5^S_1_, ^2,5^B, B_3,O_, ^1^S_3_, ^1,4^B, and ^1^S_5_ GlcA ring puckers (Figure 5b). These ring puckers all place one of these glycosidic linkage oxygen atoms in the equatorial position and the other in the axial position. For these ring puckers, the O-C_1_ and C_4_-O bond vectors are approximately perpendicular, which results in a kink in the polymer chain, and can reduce end-to-end distance even when the remainder of the polymer is fully extended (Figure 5b).

**Figure 6:**
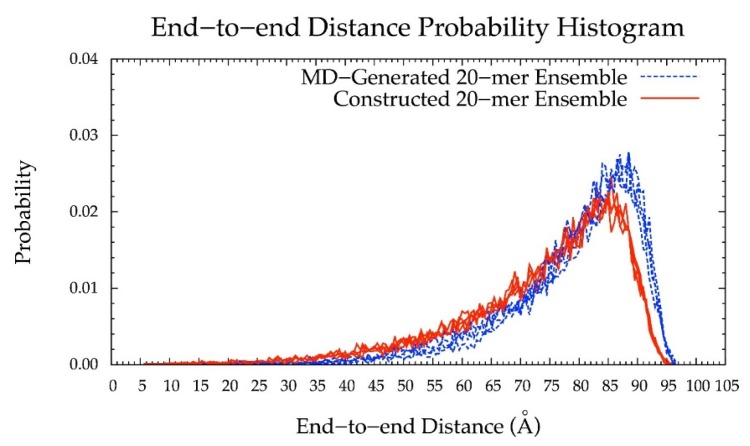
End-to-end distance probability distribution of MD-generated (blue dashed lines) and constructed (red solid lines) 20-mer ensembles; each type of ensemble includes four sets of 10,000 conformations; probabilities were calculated for end-to-end distances sorted into 0.5 Å bins.

As such, the final version of the construction algorithm uses MD-generated ring conformations instead of default force-field topology geometries. As an added benefit, this approach includes not only MD-generated ring dihedral angles but also bond lengths and angles. Using this finalized version of the algorithm, the peak in the end-to-end distance histogram for the constructed ensemble was shifted left compared to that resulting from force-field topology ring geometries (Figure 6 vs. Figure S1) and much more closely matches the reference MD-generated ensemble data. This finding shows the importance of accounting for ring flexibility in constructing chondroitin glycosaminoglycan polymer conformations similar to those sampled in all-atom explicit-solvent MD simulations. Of note, the radius of gyration was also analyzed as a function of end-to-end distance in MD-generated and constructed ensembles after minimization (Figure S2a,b). These results showed that the radius of gyration is highly correlated with end-to-end distance in both MD-generated and constructed ensembles.

While polymer kinks from ring puckering can lead to the shortening of polymer end-to-end distances, they are not required to achieve this. For 20-mers, flexibility in the glycosidic linkages even with ^4^C_1_ ring puckering in all constituent monosaccharides can be sufficient to produce compact conformations (Figure 5c). Furthermore, because of the flexibility in the glycosidic linkages flanking non-^4^C_1_ ring puckers, polymer kinks from ring puckering do not always lead to compact conformations. Thus, the leftward shift in the end-to-end distance histogram upon the inclusion of non-^4^C_1_ ring puckers supplements glycosidic linkage flexibility in yielding compact conformations.

### 3.3. Treating Glycosidic Linkage and Ring Pucker Geometries as Independent Variables

**Figure 7:**
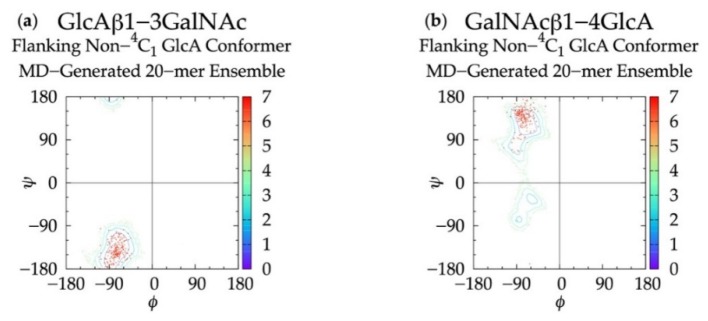
∆*G*(*ϕ*, *ψ*) plots for glycosidic linkages flanking non-^4^C_1_ GlcA conformers in non-sulfated chondroitin 20-mer MD-generated ensembles: (**a**) GlcAβ1-3GalNAc and (**b**) GalNAcβ1-4GlcA.

To determine if linkage geometries and ring deformations are interdependent, individual glycosidic linkage Δ*G*(*ϕ*, *ψ*) plots (Figure S3) were created (as opposed to the aggregate data Δ*G*(*ϕ*, *ψ*) plots in Figure 3) and no distinguishing patterns emerged. These per-linkage glycosidic linkage data were also examined in the context of C-P plots of adjacent rings (Figure S4), for which there were also no distinguishing patterns. Additionally, *ϕ* and *ψ* values in linkages flanking GlcA rings not in a ^4^C_1_ chair conformation were checked. For each linkage type, these conformations were all centered about the global Δ*G*(*ϕ*, *ψ*) minima for the aggregate data (Figure 3), and 99.96% of conformations fell within the basin extending to Δ*G*(*ϕ*, *ψ*) = +2 kcal/mol (Figure 7). Furthermore, different types of non-^4^C_1_ chair conformers did not have unique flanking linkage geometries. As no connection between linkage and ring conformations was observed in this analysis of the MD data, each linkage conformation and ring pucker was treated independently in the construction algorithm.

### 3.4. Handling Non-physical Constructed Conformations

**Figure 8:**
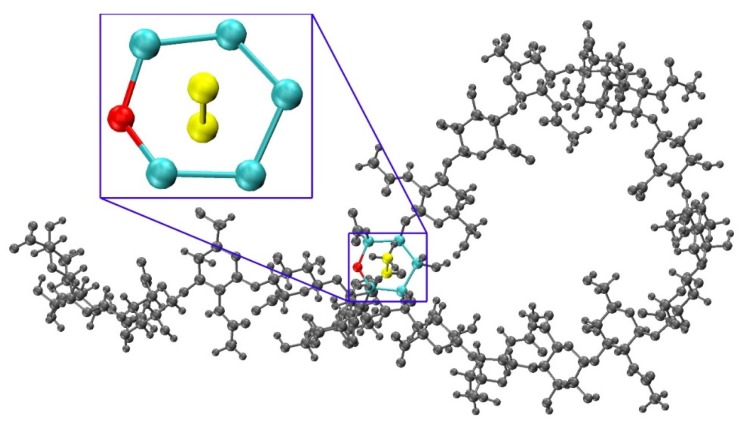
Constructed 20-mer conformation with a GlcA ring pierced by a GalNAc C-CT bond and a close up panel showing atoms involved in the ring pierce; *E*_b_ = 787.7 kcal/mol, fully-extended 20-mer post-minimization *E*_b_ = 29.6 kcal/mol, ∆*E*_b_ = 758.1 kcal/mol (Table S2).

To determine a criterion to exclude non-physical conformations from constructed ensembles, energy minimization with restrained endocyclic ring and glycosidic linkage dihedrals was performed on each conformation, and post-minimization bond potential energy and bond length probability distributions were analyzed. Conformations with outlying bond energies and abnormally long bonds may point to ring piercing (Figure 8) that cannot be fixed by minimization. To confirm this possibility, the post-minimization conformations with bond energies greater than that of the fully-extended 20-mer conformation after minimization were visualized. As anticipated, among these conformations, most with outlying total bond energies contained pierced rings which were not resolved by minimization. For each of the 12 20-mer conformations with a pierced ring, the difference between the post-minimization bond energy and that of the fully-extended 20-mer conformation is greater than the predicted energy change caused by bond distortions of that pierced ring (Table S2). The six conformations with outlying bond energies that did not contain pierced rings had kinks that resulted in bond length and bond angle distortions in glycosidic linkages that were nearly overlapping even after minimization. Of note, those conformations with bond energies that were not outlying were fully extended. These findings motivated using a bond potential energy cutoff in the construction algorithm. As stated previously, applying this cutoff resulted in 18 conformations being excluded during creation of the 40,000-member constructed ensemble. The resulting constructed ensemble contains no outlying bond lengths (Figure S5).

Dihedral angles before and after energy minimization were compared by analyzing glycosidic linkage Δ*G*(*ϕ*, *ψ*) (Table 1 and Figure 3c–f), monosaccharide ring C-P parameters (Figure 4c–f), and the change in *ϕ* and *ψ* dihedral angles due to minimization. All Δ*G*(*ϕ*, *ψ*) and C-P plots after energy minimization match those from before energy minimization and 99.6% of all *ϕ* and *ψ* dihedral angle differences before and after minimization are within 4° (Figure S6). This confirms that dihedrals do not undergo any major changes during minimization. Additionally, differences in end-to-end distance before and after minimization were calculated and the maximum change is 2.13 Å with 99.9% of changes under 0.5 Å, confirming that overall backbone conformation does not change as a result of minimization. According to these results, the selected bond potential energy cutoff and restraint scheme during minimization give conformations with little deviation from the initial constructed conformations before minimization.

### 3.5. Internal Validation on 10-mers

**Figure 9:**
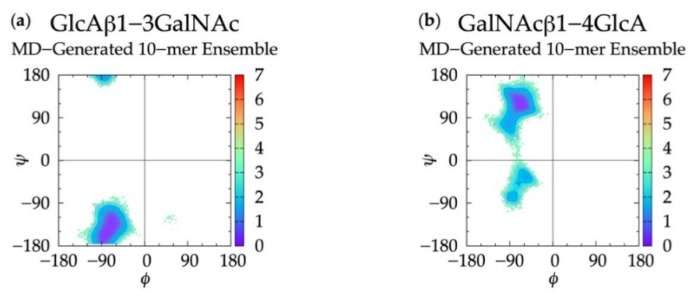
∆*G*(*ϕ*, *ψ*) plots for each glycosidic linkage in non-sulfated chondroitin 10-mer MD-generated ensembles: (**a**) GlcAβ1-3GalNAc and (**b**) GalNAcβ1-4GlcA.

To further validate the algorithm and test the use of conformational parameters from MD-generated 20-mer ensembles to construct polymers of variable length, we constructed non-sulfated chondroitin 10-mer ensembles and compared them to MD-generated 10-mer ensembles. All linkage and most ring conformations in MD-generated 10-mer (Figure 9 and Figure S7) and 20-mer ensembles matched (Figure 3a,b and Figure 4a,b), with the exception of non-^4^C_1_ GlcA rings which were not sampled in 10-mer simulations. This finding, combined with the report from NMR and force-field studies that GlcA skew-boat and boat conformations are negligible in non-sulfated chondroitin mono- and oligosaccharides [50,98], suggests that these GlcA conformations may result from intramolecular interactions in longer GAG polymers.

**Figure 10:**
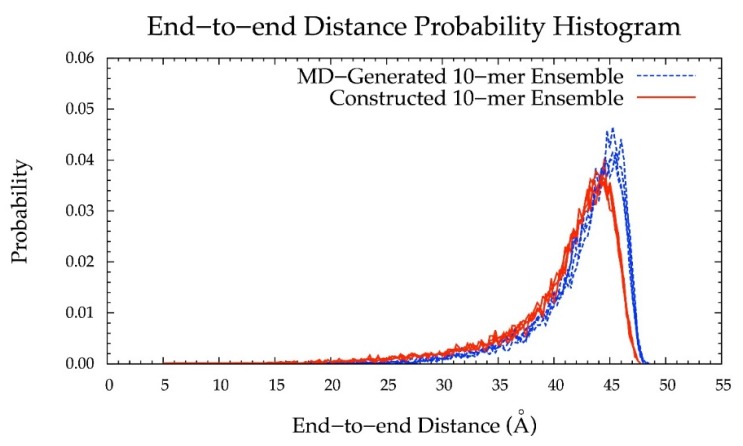
End-to-end distance probability distribution of MD-generated (blue dashed lines) and constructed (red solid lines) 10-mer ensembles; each type of ensemble includes four sets of 10,000 conformations; probabilities were calculated for end-to-end distances sorted into 0.25 Å bins.

Additionally, the end-to-end distance distributions of constructed and MD-generated 10-mer ensembles matched with minimal difference in the most probable end-to-end distance (Figure 10 and Table 2). Further, the radius of gyration is highly correlated with end-to-end distance in both MD-generated and constructed ensembles (Figure S2c,d). Of note, the end-to-end distance distributions of MD-generated 20-mer ensembles more closely matched those of 20-mer ensembles constructed using MD-generated 20-mer conformations (Figure 6) than those of 20-mer ensembles constructed using MD-generated disaccharide conformations (Figures S8 and S9). Together, these findings suggest that MD-generated 20-mer conformational parameters are ideal for constructing chondroitin polymers of different lengths.

### 3.6. Application to Longer Chondroitin Polymers

**Figure 11:**
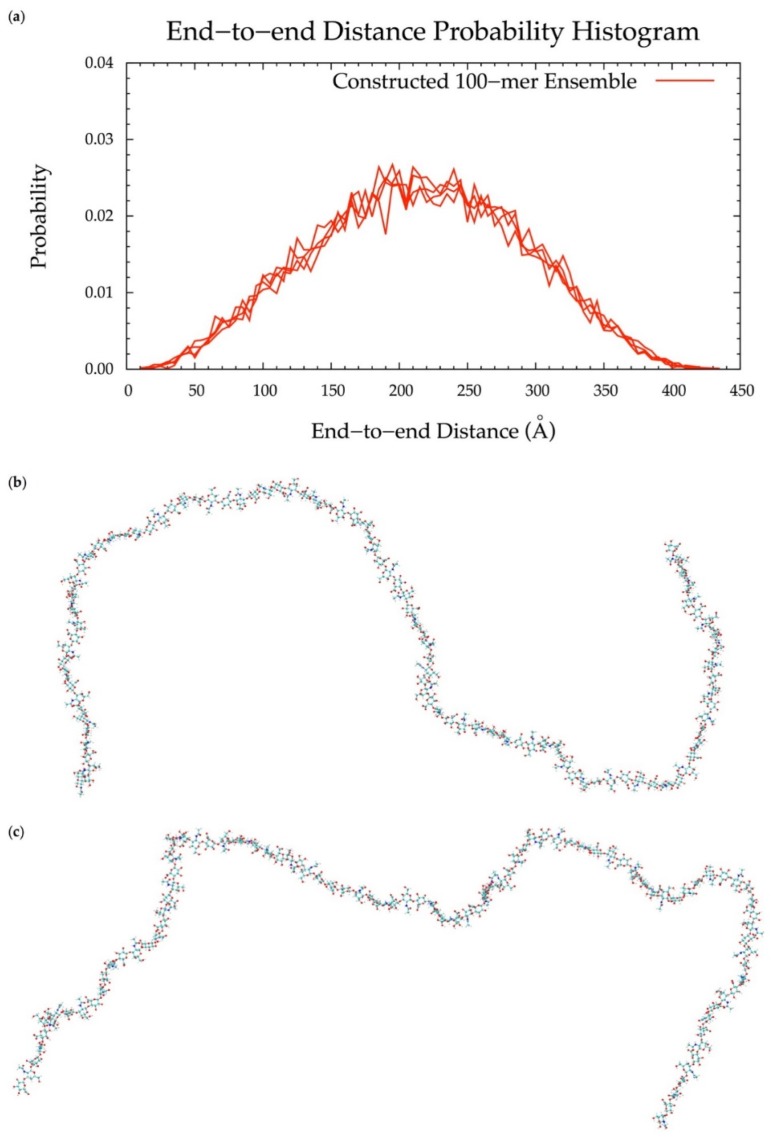
(**a**) End-to-end distance probability distribution of constructed 100-mer ensemble; includes four sets of 10,000 conformations; probabilities were calculated for end-to-end distances sorted into 5 Å bins. (**b**,**c**) Snapshots of the non-sulfated chondroitin 100-mer having the most-probable end-to-end distance (225 Å in both snapshots) from constructed ensembles.

**Figure 12:**
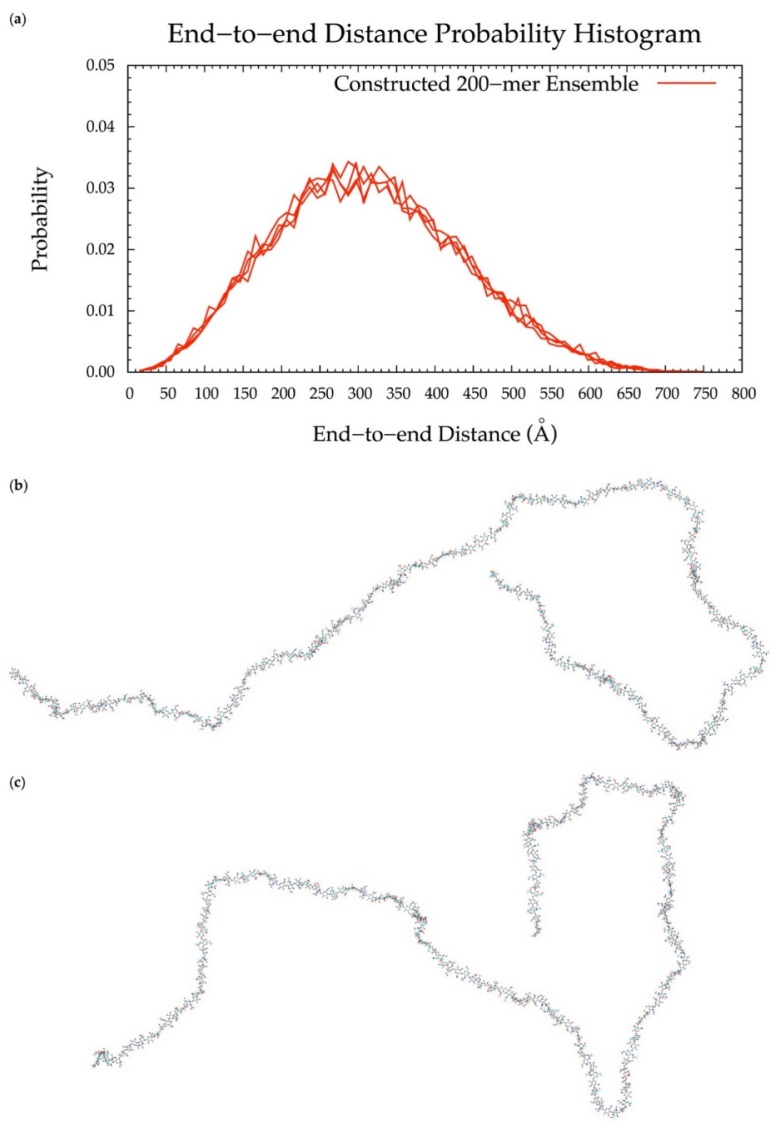
(**a**) End-to-end distance probability distribution of constructed 200-mer ensemble; includes four sets of 10,000 conformations; probabilities were calculated for end-to-end distances sorted into 10 Å bins. (**b**,**c**) Snapshots of the non-sulfated chondroitin 200-mer having the most-probable end-to-end distance (300 Å in both snapshots) from constructed ensembles.

To implement the algorithm in the construction of conformational ensembles of non-sulfated chondroitin polymers of biologically-relevant chain lengths, chondroitin 100-mer and 200-mer ensembles were constructed and the end-to-end distance (Figure 11 and Figure 12), radius of gyration (Figure S2e,f), and bond potential energy distributions (Figure S10c,d) were examined. The skewness of the end-to-end distance distributions shifts toward the right with increasing polymer length. This stands to reason, as there is a greater chance of folding with longer chains. This also explains why there are more frames excluded from these ensembles (i.e., 457 and 1407 frames excluded from the 100-mer and 200-mer ensembles, respectively). Bond potential energy distributions have similarly-shaped curves in all polymer lengths (Figure S10) and energy values increase linearly as a function of atom count (Figure S11). These results suggest that 100-mer and 200-mer conformational ensembles constructed using our algorithm (Figure 11 and Figure 12) are reasonable predictions of biological conformations given their high number of degrees of freedom.

## 4. Conclusions

With (1), all bond, bond angle, and dihedral angle conformational parameters from MD incorporated into the algorithm, (2) monosaccharide rings and glycosidic linkages treated independently, (3) energy minimization performed on each constructed conformation, and (4) a bond potential energy cutoff applied, end-to-end distance probability distributions from constructed and MD-generated ensembles match with minimal differences in most probable end-to-end distances (Table 2 and Figure 6, Figure 10, Figure 11 and Figure 12) suggesting that our algorithm produces conformational ensembles that mimic the backbone flexibility seen in MD simulations of non-sulfated chondroitin polymers.

Our program is also valuable for its efficiency. For example, the fully-solvated chondroitin 20-mer system contains ~191,000 atoms and took about one month to simulate. It took only about 12.5 h to construct the 20-mer conformational ensembles using our algorithm, in which all end-to-end distances, radii of gyration, bond lengths, dihedral angles, monosaccharide ring PDB files for C-P analysis of every tenth frame, and bond and system potential energies before and after minimization, C-P parameters of every GlcA ring in every frame, and PDBs of all conformations with bond energies greater than that of the fully-extended 20-mer (including conformations with pierced rings) are written. The fully-solvated chondroitin 10-mer system contains ~36,000 atoms and took about five days to simulate. The 10-mer ensembles were constructed using our algorithm in about 40 min and energies, dihedral angles, end-to-end distances, and radii of gyration after minimization, C-P parameters of every GlcA ring in every frame, and PDBs of all conformations with bond energies greater than that of the fully-extended 10-mer were written. Fully-solvated chondroitin 100-mer and 200-mer systems would contain ~3,370,000 atoms and ~75,450,000 atoms, respectively. Systems of this magnitude are not feasible to simulate with current computational resources but if they could be simulated, they would take on the order of years to complete. Construction of the chondroitin 100-mer and 200-mer conformational ensembles using our algorithm took about four and nine hours, respectively, and the algorithm produced the same output data types as for the 10-mer (n.b.: these timings are less than the 20-mer timing above because of all of the additional output written to disk for analysis in the case of the 20-mer ensemble construction).

In conclusion, our algorithm, incorporating glycosidic linkage and monosaccharide ring conformations from the MD simulation of non-sulfated chondroitin 20-mers, can be used to efficiently generate conformational ensembles of non-sulfated chondroitin polymers of arbitrary length. We are investigating the applicability of this approach to various sulfo-forms of CS and different types of GAGs, including hyaluronan (HA), dermatan sulfate (DS), heparan sulfate (HS), and keratan sulfate (KS). Given the variability and complexity of GAGs, as well as existing barriers to the experimental characterization of the three-dimensional conformational properties of GAGs of lengths relevant in the context of PGs, there are currently very few efforts to target GAGs. We anticipate that the presented algorithm, combined with experimental data on PG core proteins and conformational analysis of the linker tetrasaccharide [48,52,102,103,104], may provide a useful means of generating atomic-resolution three-dimensional models of full PGs. The algorithm could also be used to model full GAG–protein complexes, which may provide insights into potential interactions between multiple biomolecules within a single GAG complex. The ability to model these complex biomolecules would be a key step towards improving understanding of GAG bioactivity, assessing the druggability of GAGs, designing agonists or antagonists to treat disease, and developing diagnostic tools. Thus, this methodology may open a new avenue into disease modulation.

## Figures and Tables

**Table 1:** Glycosidic Linkage Dihedrals (*ϕ*, *ψ*) ^1^ and Free Energy (Δ*G*(*ϕ*, *ψ*) kcal/mol) Minima.

	MD-Generated 20-mer Ensemble				Constructed 20-mer Ensemble (Before Energy Minimization)				Constructed 20-mer Ensemble (After Energy Minimization)			
	GlcAβ1-3 GalNAc		GalNAcβ1-4 GlcA		GlcAβ1-3 GalNAc		GalNAcβ1-4 GlcA		GlcAβ1-3 GalNAc		GalNAcβ1-4 GlcA	
Min	*ϕ, ψ*	Δ*G*	*ϕ, ψ*	Δ*G*	*ϕ, ψ*	Δ*G*	*ϕ, ψ*	Δ*G*	*ϕ, ψ*	Δ*G*	*ϕ, ψ*	Δ*G*
**I**	−81.25°, −153.75°	0.00	−66.25°, 116.25°	0.00	−76.25°, −148.75°	0.00	−66.25°, 116.25°	0.00	−81.25°, −153.75°	0.00	−68.75°, 121.25°	0.00
**II**			−58.75°, −33.75°	1.57			−61.25°, −33.75°	1.54			−58.75°, −33.75°	1.57
**II’**			−86.25°, −73.75°	1.80			−86.25°, −73.75°	1.71			−86.25°, −78.75°	1.73

^1^*ϕ*, *ψ* dihedral angles were sorted into 2.5° bins.

**Table 2:** Most Probable End-to-End Distances (*d*) in MD-Generated and Constructed Ensembles ^1^.

	20-mer Ensembles			10-mer Ensembles		
	MD-Generated *d* (Å)	Constructed *d* (Å)	% Difference	MD-Generated *d* (Å)	Constructed *d* (Å)	% Difference
**Run 1**	88.5	83.0		45.25	44.50	
**Run 2**	88.5	85.5		45.25	43.50	
**Run 3**	86.0	85.0		45.50	44.50	
**Run 4**	86.5	85.0		45.25	44.25	
**All ^2^**	88.5	85.0	4.03%	45.25	44.50	1.671%

^1^ Probabilities were calculated for end-to-end distances sorted into 0.5 Å bins for the 20-mer ensembles and 0.25 Å bins for the 10-mer ensembles. ^2^ All = end-to-end distance distribution aggregated across all four runs.

## Supplementary Materials

The following are available online https://www.mdpi.com/2218-273X/10/4/537/s1, Table S1: Comparison to Observed Literature Values of Glycosidic Linkage Dihedrals (*ϕ*, *ψ*) in Non-Sulfated Chondroitin, Table S2: Bond Energies of Constructed Chondroitin 20-mer Conformations with Pierced Rings, Figure S1: End-to-end distance probability distribution of 20-mer ensembles generated by MD and an early version of the construction algorithm which applied glycosidic linkage geometries from MD-generated 20-mer ensembles, Figure S2: Scatterplots of radius of gyration as a function of end-to-end distance in MD-generated and constructed chondroitin ensembles, Figure S3: Δ*G(**ϕ,**ψ*) plots for each glycosidic linkage in the chondroitin 20-mer from MD-generated ensembles, Figure S4: Cremer-Pople plots for each monosaccharide ring in the chondroitin 20-mer from MD-generated ensembles, Figure S5: Probability histograms of bond lengths for each type of bond in the chondroitin 20-mer from MD-generated and constructed ensembles, Figure S6: Probability histogram showing changes in glycosidic linkage *ϕ* and *ψ* dihedral angles during energy minimization in constructed 20-mer ensembles, Figure S7: Cremer-Pople plots of GalNAc and GlcA in MD-generated chondroitin 10-mer ensembles, Figure S8: End-to-end distance probability distribution of chondroitin 20-mer ensembles generated by MD and an early version of the construction algorithm which applied glycosidic linkage geometries from MD-generated non-sulfated chondroitin disaccharide ensembles, Figure S9: Δ*G*(*ϕ*,*ψ*) plots for GlcAβ1-3GalNAc and GalNAcβ1-4GlcA glycosidic linkages in MD-generated non-sulfated chondroitin disaccharide ensembles and 20-mer ensembles constructed using glycosidic linkage dihedral probabilities from these MD-generated disaccharide ensembles, Figure S10: Bond energy distribution probability histograms from constructed 10-, 20-, 100-, and 200-mer ensembles, Figure S11: Average bond energies as a function of polymer length.
