# Supplementary material for: Efficient Construction of Atomic-Resolution Models of Non-Sulfated Chondroitin Glycosaminoglycan Using Molecular Dynamics Data
Source: Biomolecules. 2020 Apr 2;10(4):537. doi: 10.3390/biom10040537 (PMC7226628; doi:10.3390/biom10040537)
Supplement: Supplementary file 1 [file biomolecules-10-00537-s001.pdf]

Supporting Information for:

Efficient construction of atomic-resolution models of non-sulfated chondroitin glycosaminoglycan using molecular dynamics data

Elizabeth K. Whitmore [1,2], Gabriel Vesenka [1], Hanna Sihler [1], and Olgun Guvench [1,2,*]

[1] Department of Pharmaceutical Sciences, University of New England College of Pharmacy, 716 Stevens Avenue, Portland, Maine, 04103, USA; ewhitmore@une.edu; gvesenka1@une.edu; hsihler@une.edu; oguvench@une.edu

[2] Graduate School of Biomedical Science and Engineering, University of Maine, 5775 Stodder Hall, Orono, Maine, 04469, USA

* Correspondence: oguvench@une.edu; Tel.: +01-207-221-4171

Table S1. Comparison to Observed Literature Values of Glycosidic Linkage Dihedrals (ϕ, ψ) in Non-Sulfated Chondroitin

		GlcAβ1-3GalNAc				GalNAcβ1-4GlcA			
	**Min**	ϕ	**Diff** [1]	ψ	**Diff** [1]	ϕ	**Diff**	ψ	**Diff** [2]
**Biased MD-generated 2-mer Ensembles [1]** [3]	**I**	-83.75°	+2.5°	83.75°	+122.5°	-63.75°	-2.5°	-121.25°	-122.5°
	**II**					-58.75°	0°	93.75°	-127.5°
	**II′**					-83.75°	-2.5°	48.75°	-122.5°
**Unbiased MD-Generated and NMR-Validated 6-mer Ensembles [2]** [4]	**I**	-72°	-9°	108°	+98°	-73°	+7°	-117°	-127°

Diff = ($x_{(obs)}$ - x) +/- 360° where $x_{(obs)}$ = ϕ or ψ observed in our 20-mer simulations

[1] Expected difference for β1-3 = +120°

[2] Expected difference for β1-4 = -120°

[3] ϕ, ψ dihedral angles were sorted into 2.5° bins.

[4] ϕ, ψ dihedral angles were sorted into 1° bins; only global minima (I) were reported.

Table S2. Bond Energies (E_b) of Constructed Chondroitin 20-mer Conformations with Pierced Rings

**Piercing Bond**	**Pierced Ring**	**Pierce Type** [1]	E_b **(kcal/mol)**	$\Delta E_b = E_b - E_{b,cut}$ [2] **(kcal/mol)**	**Estimated** ΔE_b [3] **(kcal/mol)**
GalNAc C_5-C_6	GalNAc	Exocyclic	721.0	592.2	285.2
GalNAc C_6-O_6	GalNAc	Exocyclic	784.2	655.4	132.3
GalNAc C_6-O_6	GalNAc	Exocyclic	793.0	664.2	132.3
GalNAc C-CT	GlcA	Exocyclic	787.7	658.9	253.4
GlcA C_5-C_6	GlcA	Exocyclic	720.2	591.4	294.6
GalNAc C_3-O_3	GlcA	Linkage	715.8	587.0	224.1
GlcA C_4-O_4	GalNAc	Linkage	714.8	586.0	238.6
GlcA	GalNAc	Interlocking	981.2	852.4	725.2
GlcA	GalNAc	Interlocking	941.6	812.8	725.2
GlcA	GlcA	Interlocking	961.4	832.6	723.7
GalNAc	GalNAc	Interlocking	1053	924.2	520.3
GalNAc	GalNAc	Interlocking	1110	981.2	520.3

[1] Exocyclic: the piercing bond is an exocyclic bond not participating in a glycosidic linkage; Linkage: the piercing bond is part of a glycosidic linkage; Interlocking: the rings are interlocking (i.e. there are two piercing bonds and the estimated ΔE_b is the sum of the estimated ΔE_b of these two bonds).

[2] $E_{b,cut}$ = 128.85 kcal/mol (bond energy cutoff).

[3] Bond strain energy of this piercing bond estimated by comparing this bond energy in individual nonbonded saccharide systems with and without ring piercing.

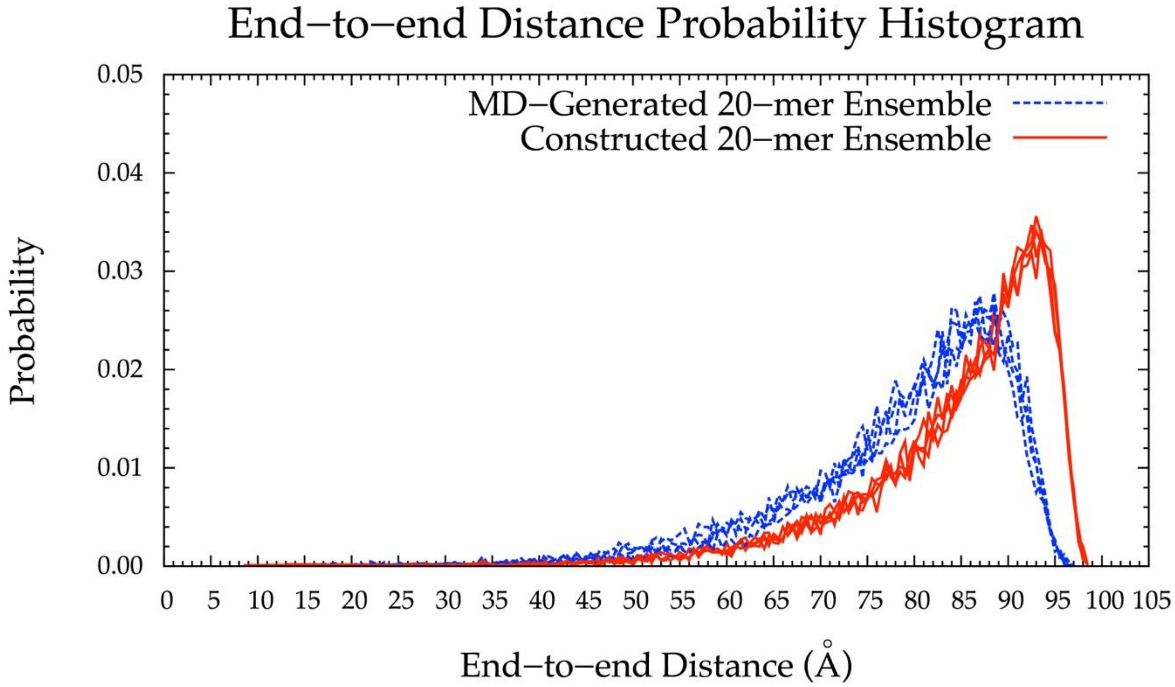

Figure S1. End-to-end distance probability distribution of 20-mer ensembles generated by MD (blue dashed lines) and an early version of the construction algorithm (red solid lines) which applied glycosidic linkage geometries from MD-generated 20-mer ensembles and standard force field geometries for all monosaccharide rings; each type of ensemble includes four sets of 10,000 conformations; probabilities were calculated for end-to-end distances sorted into 0.5 Å bins.

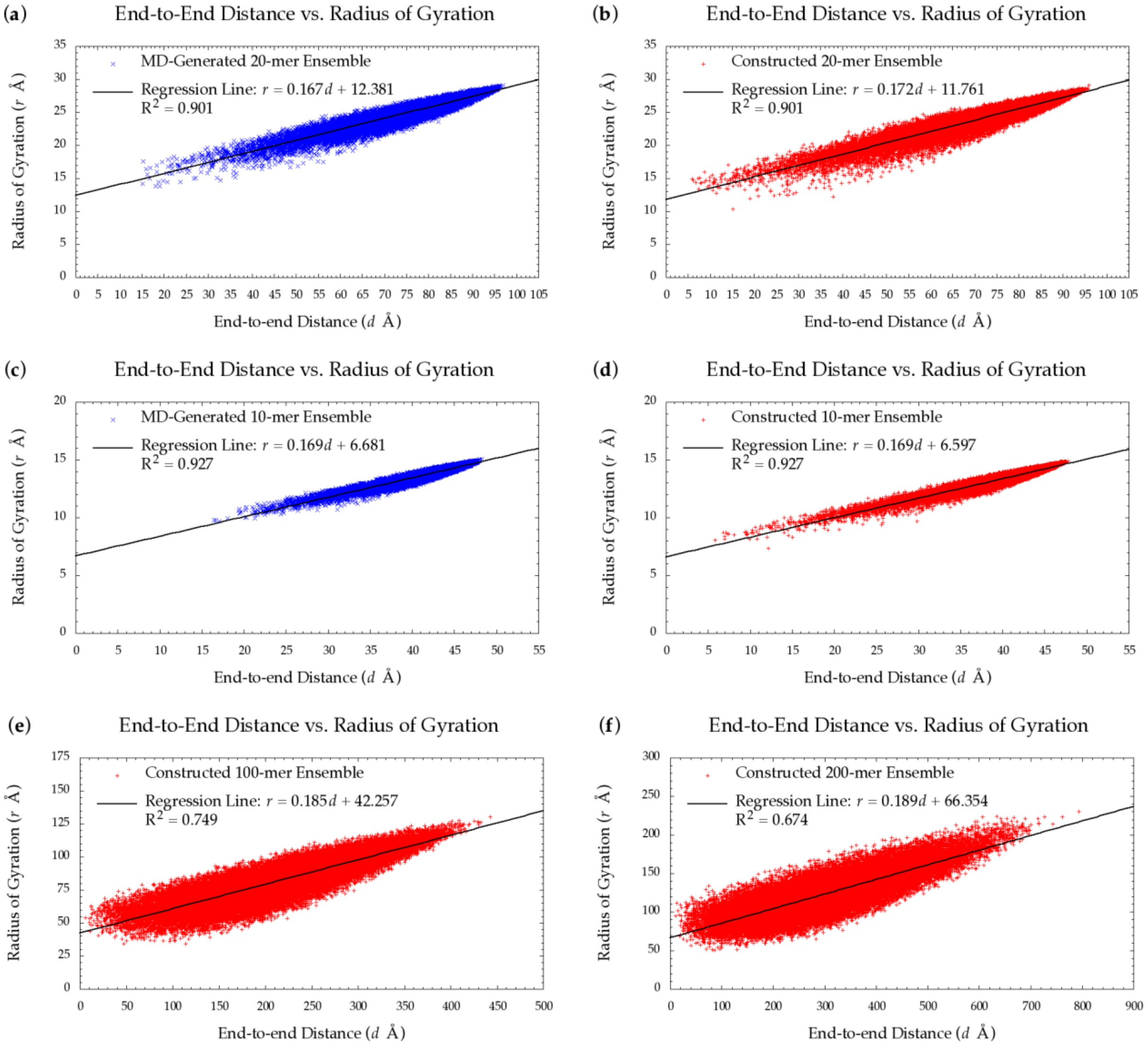

Figure S2. Scatterplots of radius of gyration as a function of end-to-end distance in MD-generated and constructed ensembles of non-sulfated chondroitin (**a**,**b**) 20-mer and (**c**,**d**) 10-mer, respectively, and constructed ensembles of the chondroitin (**e**) 100-mer and (**f**) 200-mer. Each plot has 40,000 samples and shows linear regression and R^2.

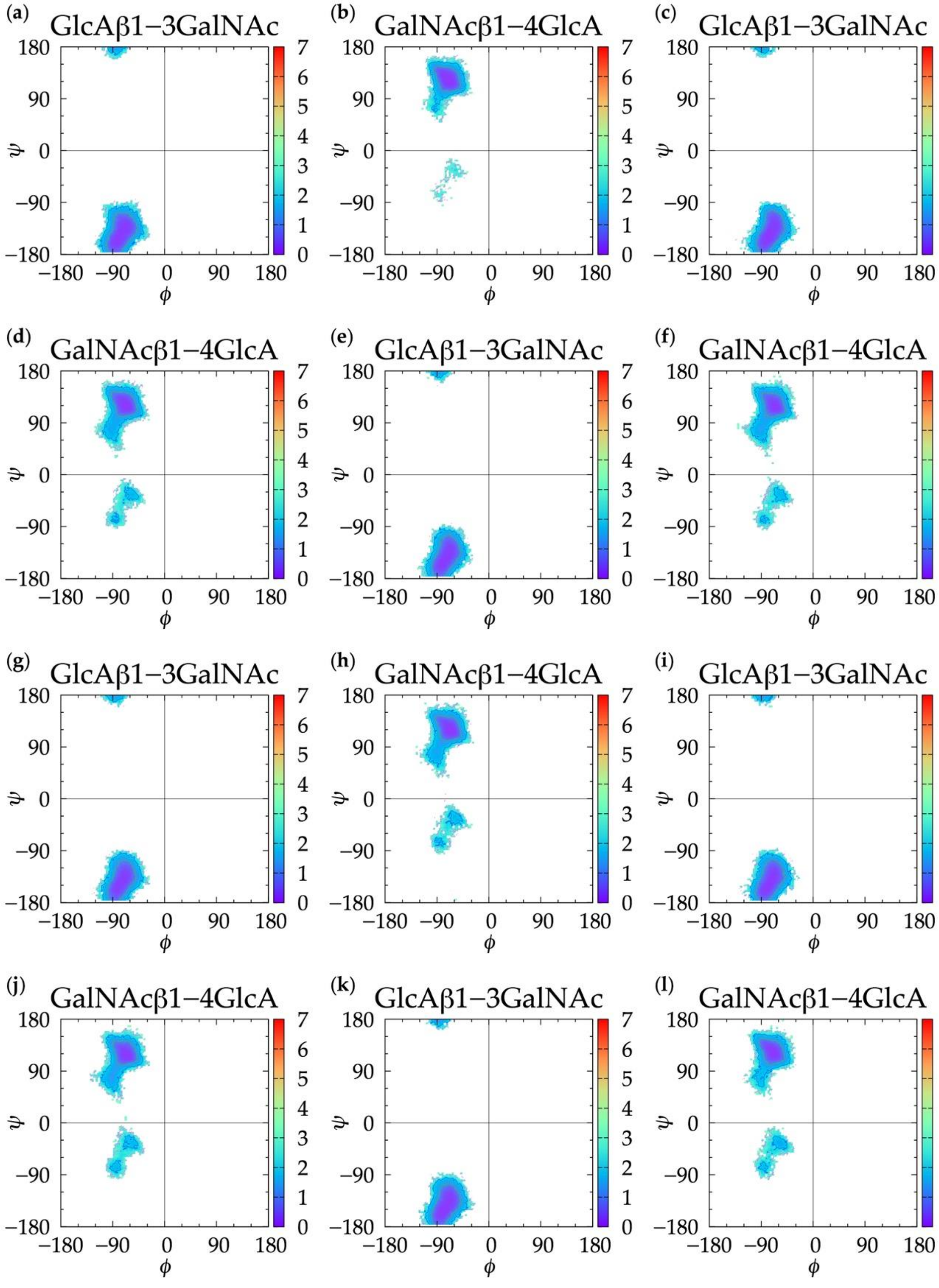
(a) GlcAβ1−3GalNAc
(b) GalNAcβ1−4GlcA
(c) GlcAβ1−3GalNAc
(d) GalNAcβ1−4GlcA
(e) GlcAβ1−3GalNAc
(f) GalNAcβ1−4GlcA
(g) GlcAβ1−3GalNAc
(h) GalNAcβ1−4GlcA
(i) GlcAβ1−3GalNAc
(j) GalNAcβ1−4GlcA
(k) GlcAβ1−3GalNAc
(l) GalNAcβ1−4GlcA
ψ
ϕ
180
90
0
−90
−180
0
1
2
3
4
5
6
7

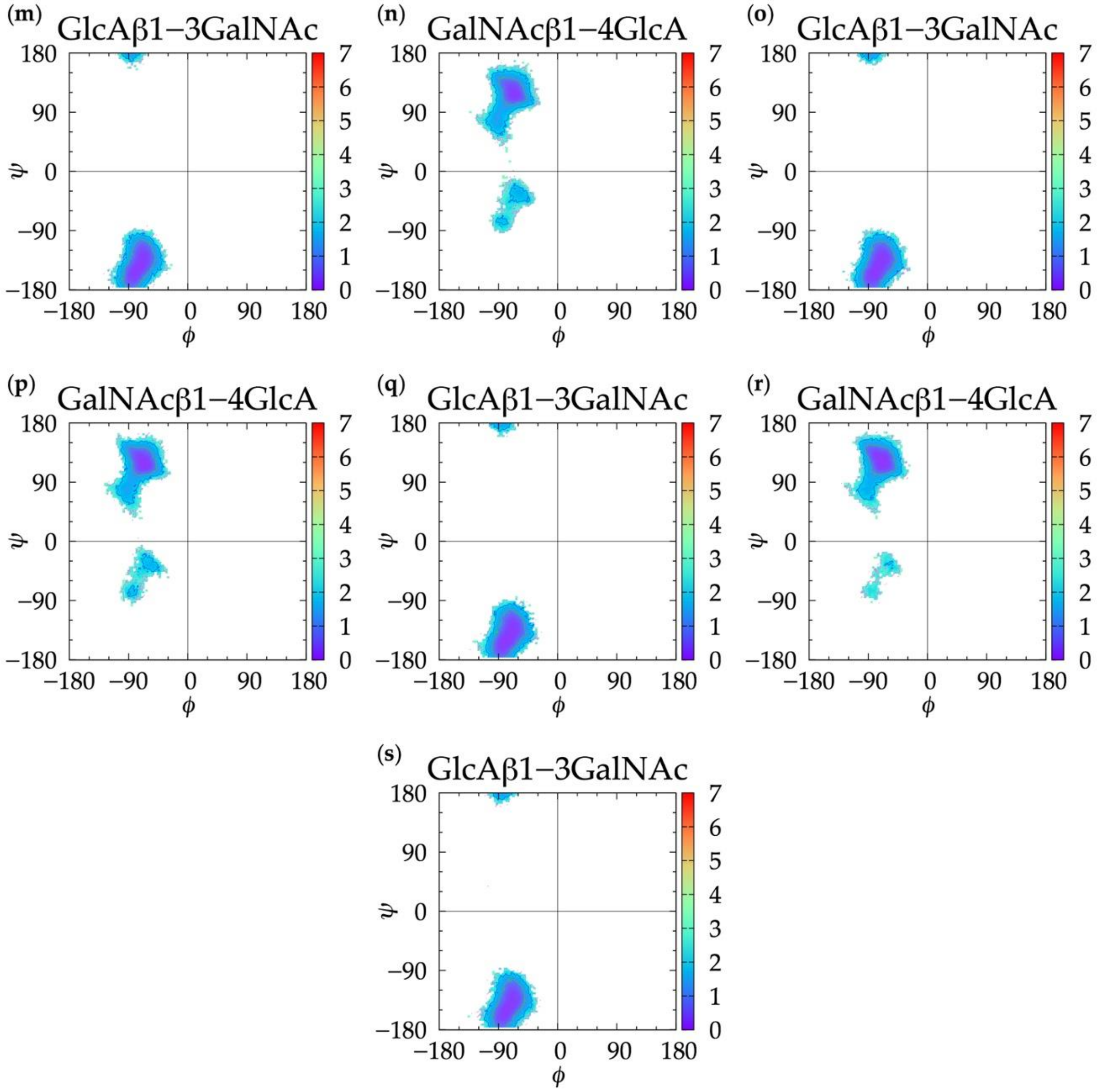

Figure S3. $\Delta G(\phi,\psi)$ plots for each glycosidic linkage in the chondroitin 20-mer from MD-generated ensembles; (**a**) GlcA2←GalNAc1, (**b**) GalNAc3←GlcA2, (**c**) GlcA4←GalNAc3, (**d**) GalNAc5←GlcA4, (**e**) GlcA6←GalNAc5, (**f**) GalNAc7←GlcA6, (**g**) GlcA8←GalNAc7, (**h**) GalNAc9←GlcA8, (**i**) GlcA10←GalNAc9, (**j**) GalNAc11←GlcA10, (**k**) GlcA12←GalNAc11, (**l**) GalNAc13←GlcA12, (**m**) GlcA14←GalNAc13, (**n**) GalNAc15←GlcA14, (**o**) GlcA16←GalNAc15, (**p**) GalNAc17←GlcA16, (**q**) GlcA18←GalNAc17, (**r**) GalNAc19←GlcA18, and (**s**) GlcA20←GalNAc19; monosaccharides are numbered from reducing to non-reducing end; ϕ, ψ separated into 2.5° bins.

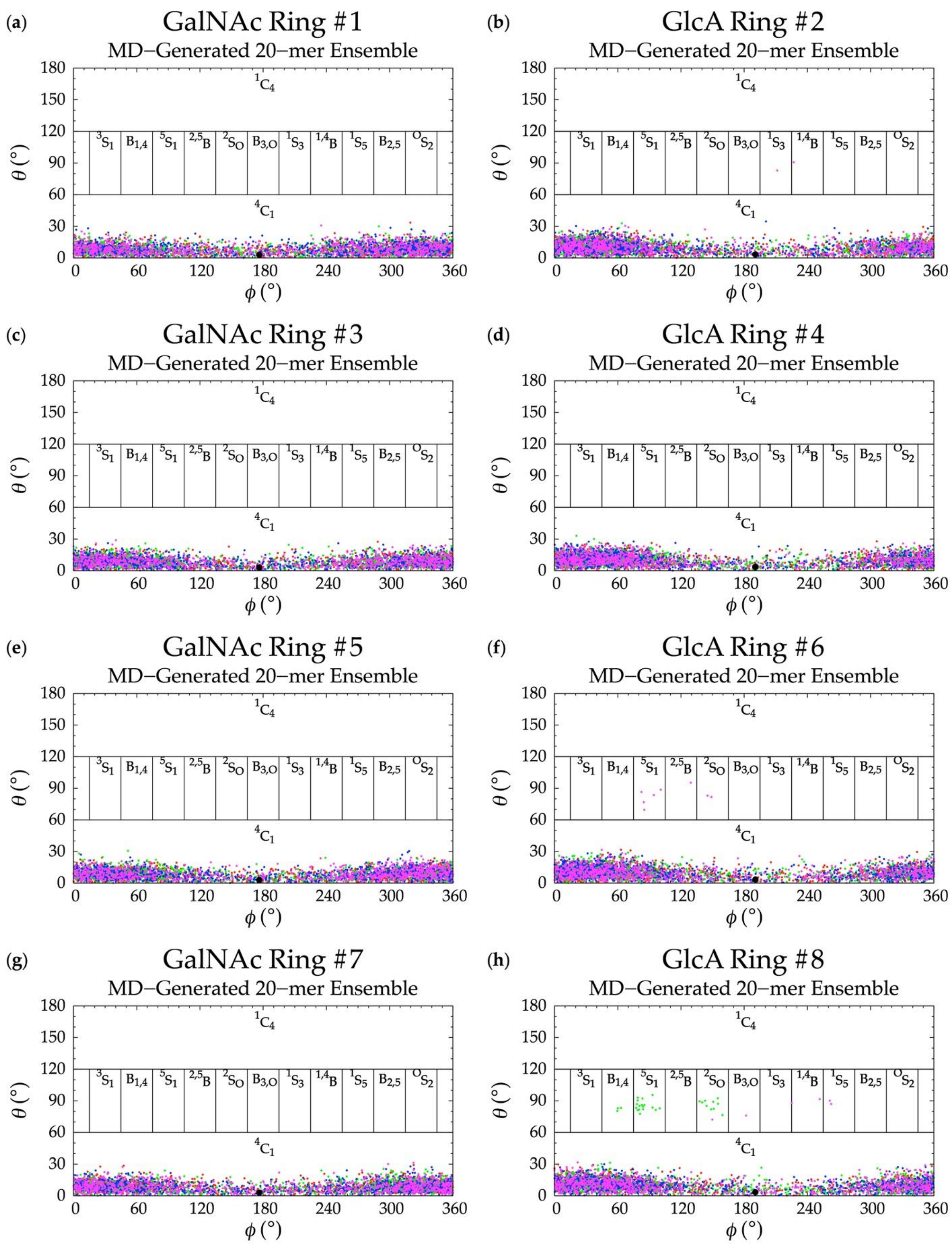
(a) GalNAc Ring #1
MD−Generated 20−mer Ensemble
(b) GlcA Ring #2
MD−Generated 20−mer Ensemble
(c) GalNAc Ring #3
MD−Generated 20−mer Ensemble
(d) GlcA Ring #4
MD−Generated 20−mer Ensemble
(e) GalNAc Ring #5
MD−Generated 20−mer Ensemble
(f) GlcA Ring #6
MD−Generated 20−mer Ensemble
(g) GalNAc Ring #7
MD−Generated 20−mer Ensemble
(h) GlcA Ring #8
MD−Generated 20−mer Ensemble
$^1C_4$
$^3S_1$ $B_{1,4}$ $^5S_1$ $^{2,5}B$ ^2S_O $B_{3,O}$ $^1S_3$ $^{1,4}B$ $^1S_5$ $B_{2,5}$ $^OS_2$
$^4C_1$
θ (°)
φ (°)

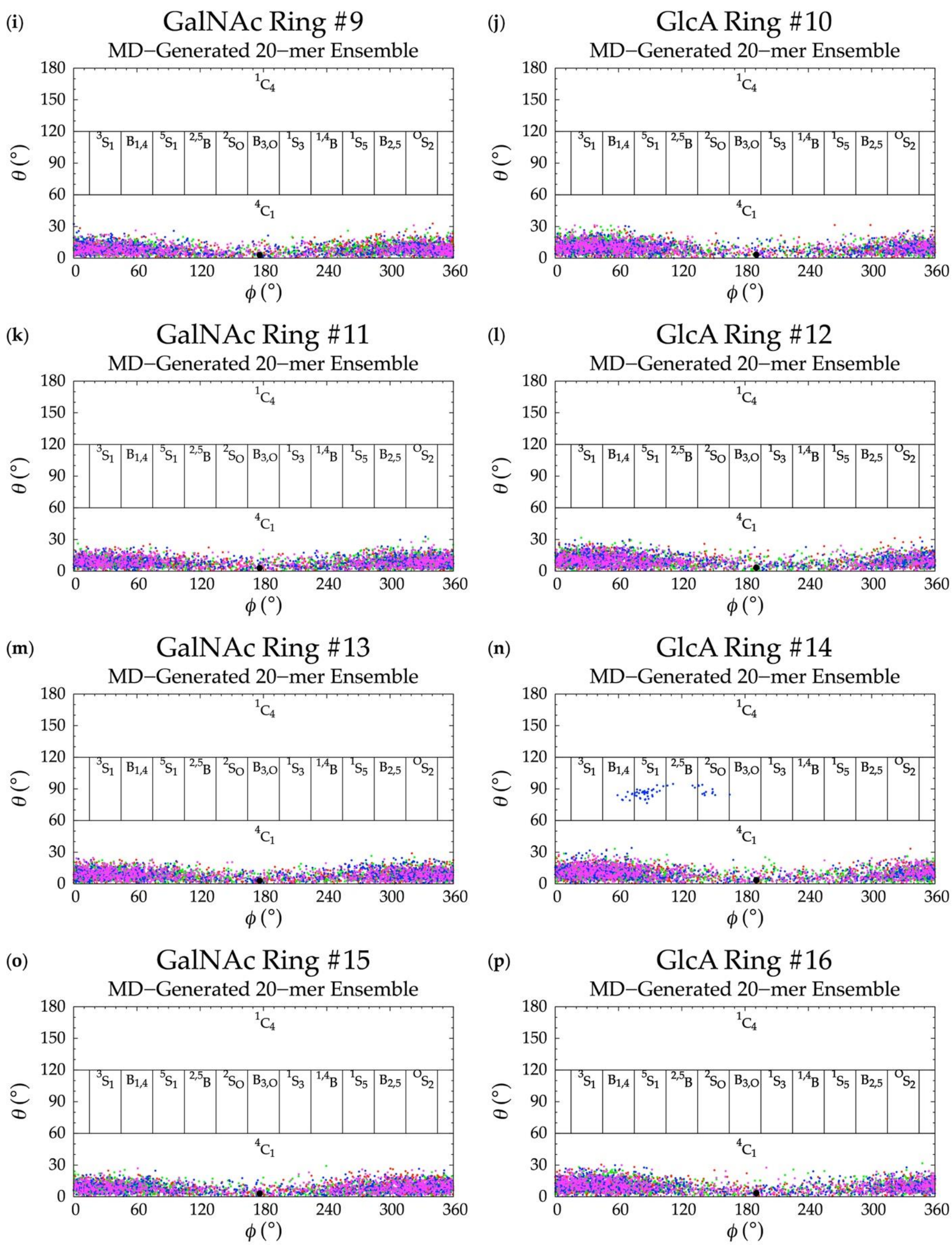

(i)
GalNAc Ring #9
MD−Generated 20−mer Ensemble
(j)
GlcA Ring #10
MD−Generated 20−mer Ensemble
(k)
GalNAc Ring #11
MD−Generated 20−mer Ensemble
(l)
GlcA Ring #12
MD−Generated 20−mer Ensemble
(m)
GalNAc Ring #13
MD−Generated 20−mer Ensemble
(n)
GlcA Ring #14
MD−Generated 20−mer Ensemble
(o)
GalNAc Ring #15
MD−Generated 20−mer Ensemble
(p)
GlcA Ring #16
MD−Generated 20−mer Ensemble
$^1C_4$
$^3S_1$ $B_{1,4}$ $^5S_1$ $^{2,5}B$ ^2S_O $B_{3,O}$ $^1S_3$ $^{1,4}B$ $^1S_5$ $B_{2,5}$ $^OS_2$
$^4C_1$
θ (°)
ϕ (°)
0 30 60 90 120 150 180
0 60 120 180 240 300 360

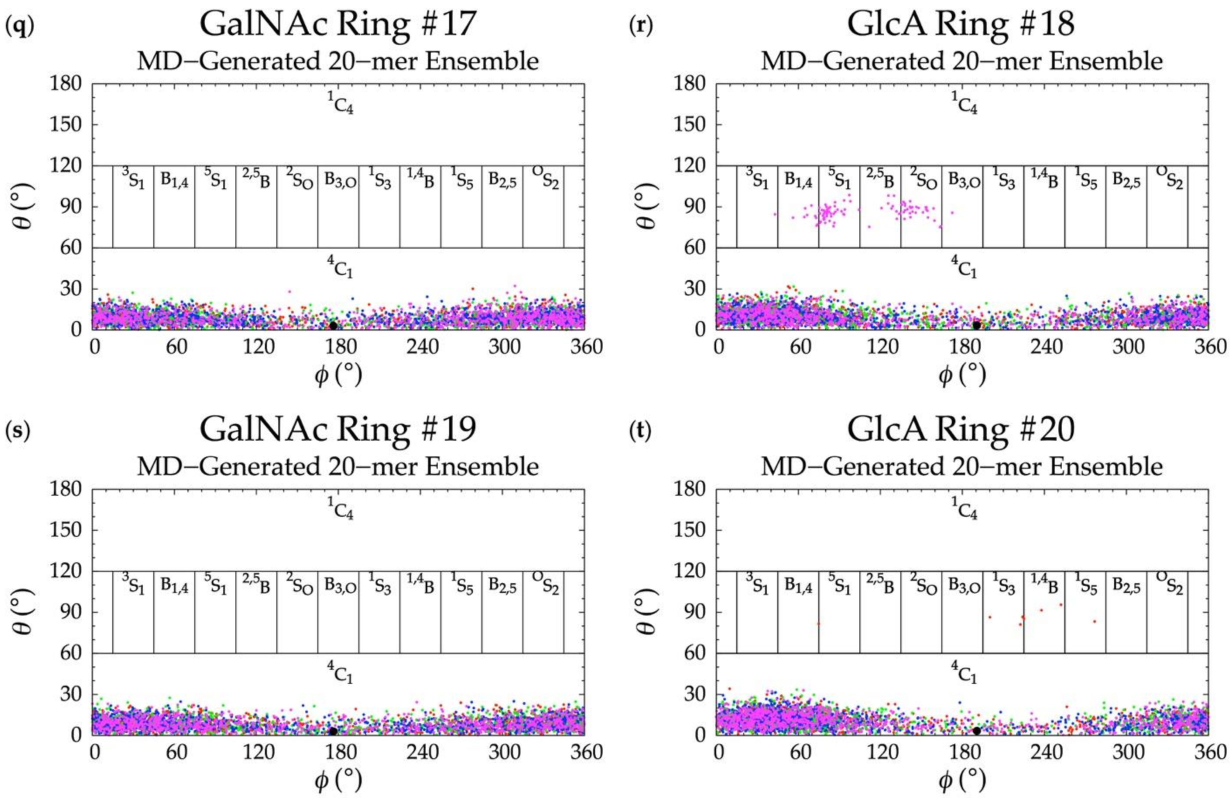

Figure S4. (**a**-**t**) Cremer-Pople plots for each monosaccharide ring in the chondroitin 20-mer from MD-generated ensembles; monosaccharides are numbered from reducing to non-reducing end; each of the 4 runs is represented by different color.

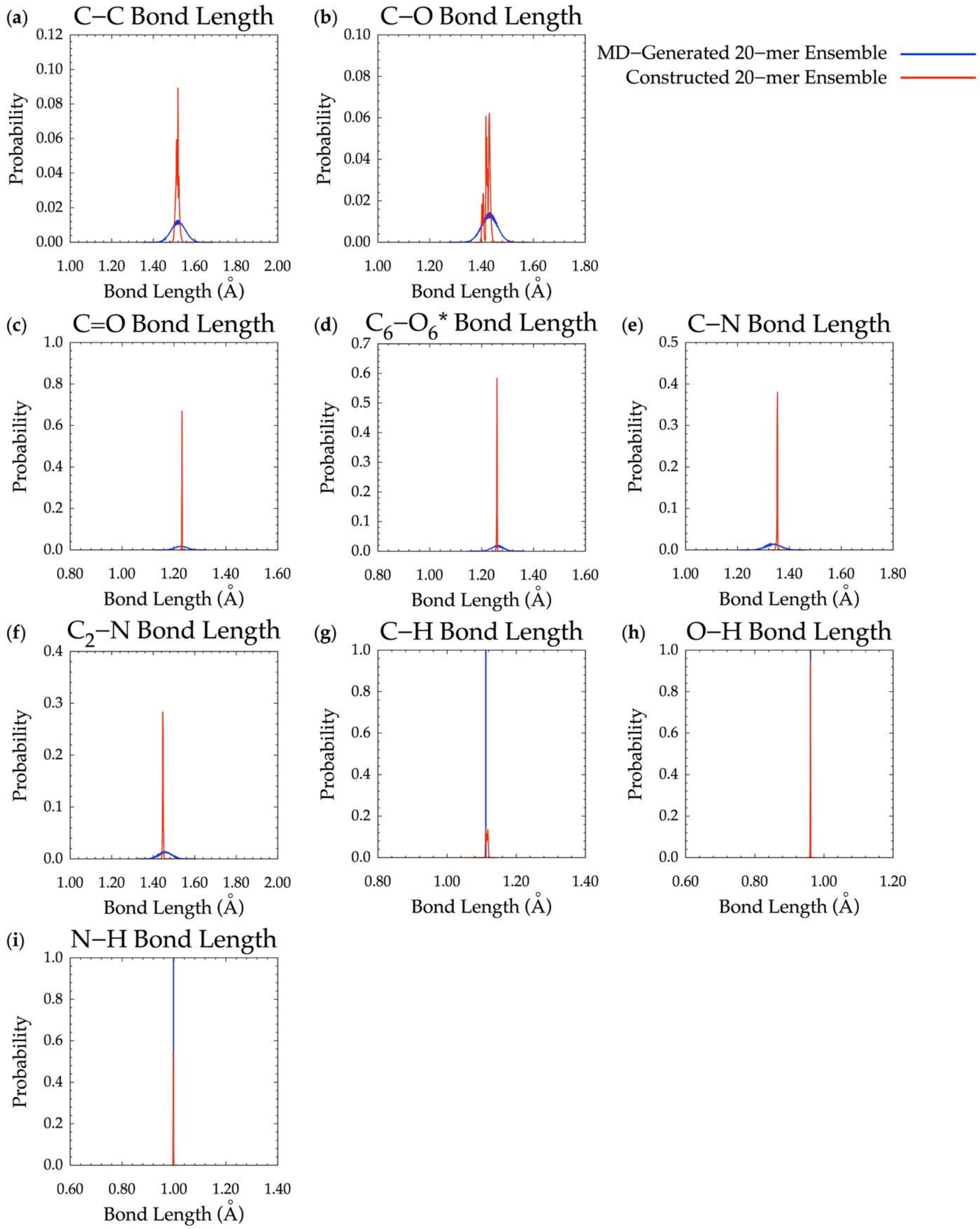

Figure S5. (**a**-**i**) Probability histograms of bond lengths for each type of bond in the chondroitin 20-mer; blue lines represent bond lengths in MD-generated ensembles (which match those in constructed ensembles before minimization) and red lines represent bond lengths in constructed 20-mer ensembles after minimization. (Note: bond lengths involving hydrogen atoms (**g**-**i**) are fixed during MD but not during minimization in the algorithm.)

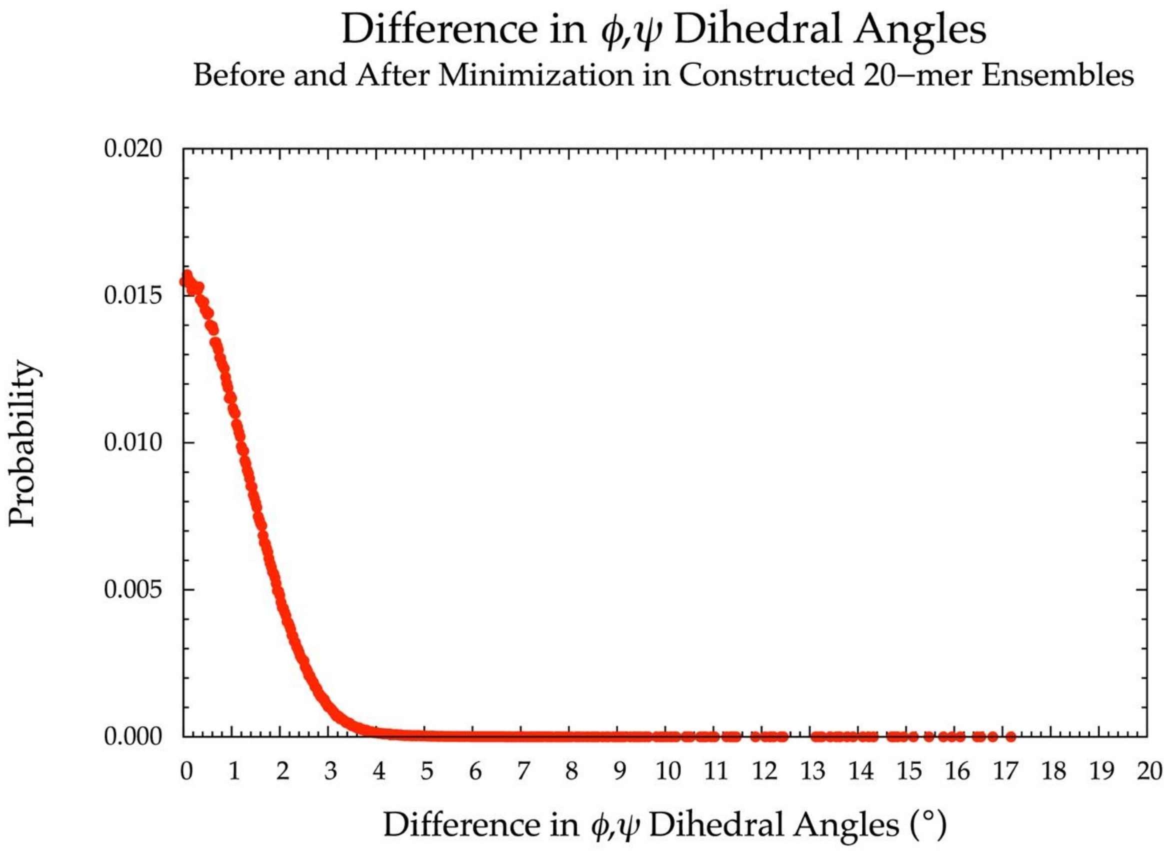

Figure S6. Probability histogram showing changes in glycosidic linkage ϕ and ψ dihedral angles during energy minimization in constructed 20-mer ensembles; 99.6% of all differences are within 4°.

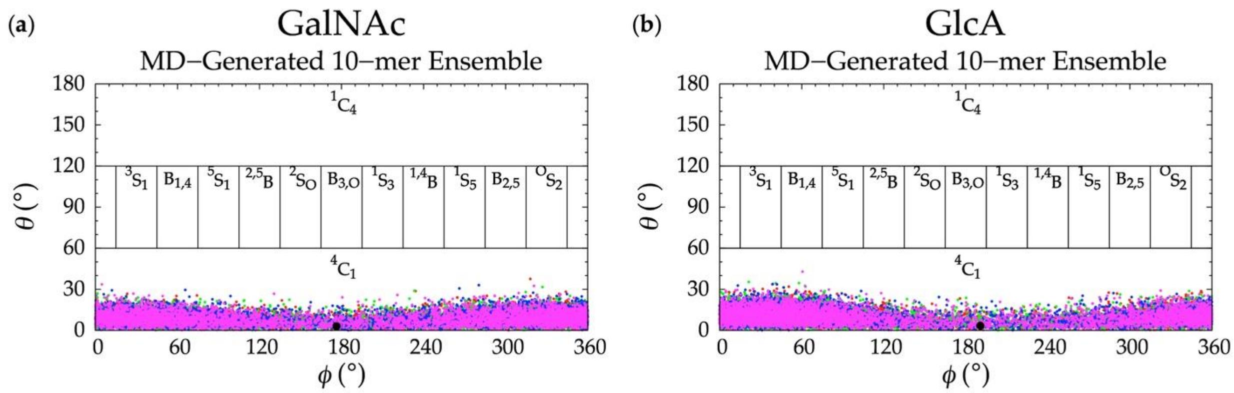

Figure S7. Cremer-Pople plots of (**a**) GalNAc and (**b**) GlcA in MD-generated chondroitin 10-mer ensembles; geometries from the four sets of each type of ensemble are represented by red, green, blue, and magenta dots, respectively and the force field geometry is represented by a black dot. Cremer-Pople parameters of all rings in every tenth snapshot from each ensemble were plotted (i.e. 5 rings * 1,000 snapshots per run * 4 runs = 20,000 parameter sets).

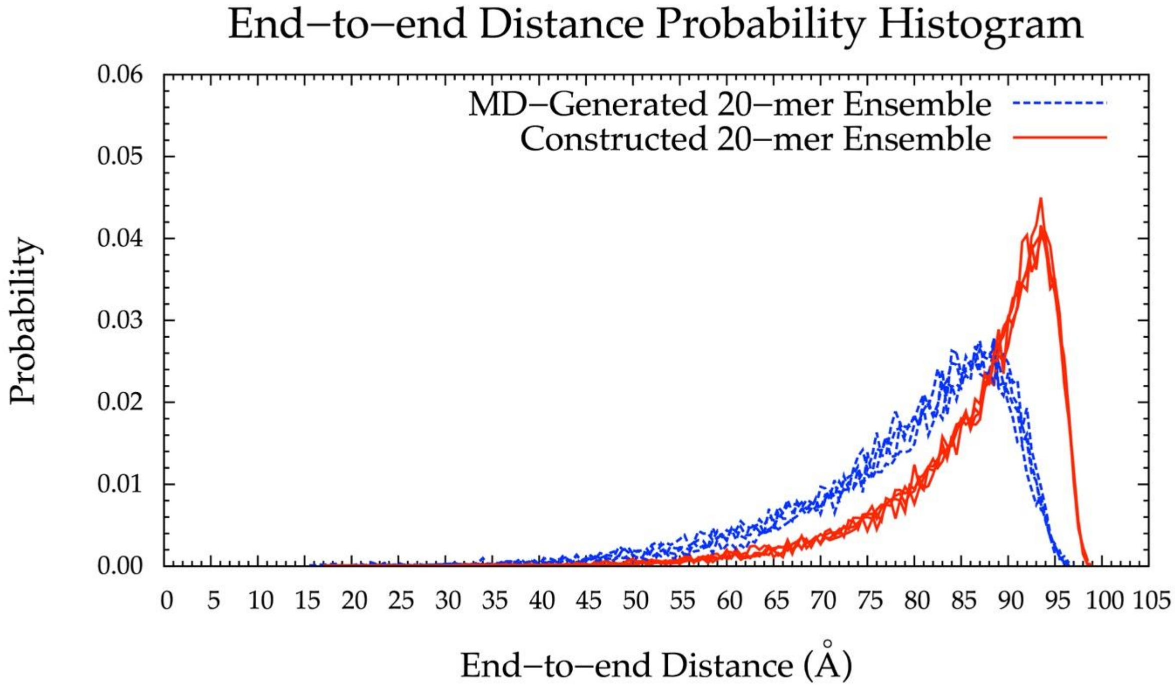

Figure S8. End-to-end distance probability distribution of chondroitin 20-mer ensembles generated by MD (blue dashed lines) and an early version of the construction algorithm (red solid lines) which applied glycosidic linkage geometries from Adaptive Biasing Force (ABF) [3,4] MD-generated ensembles of non-sulfated chondroitin disaccharides [1] and standard force field geometries for all monosaccharide rings; each type of ensemble includes four sets of 10,000 conformations; probabilities were calculated for end-to-end distances sorted into 0.5 Å bins.

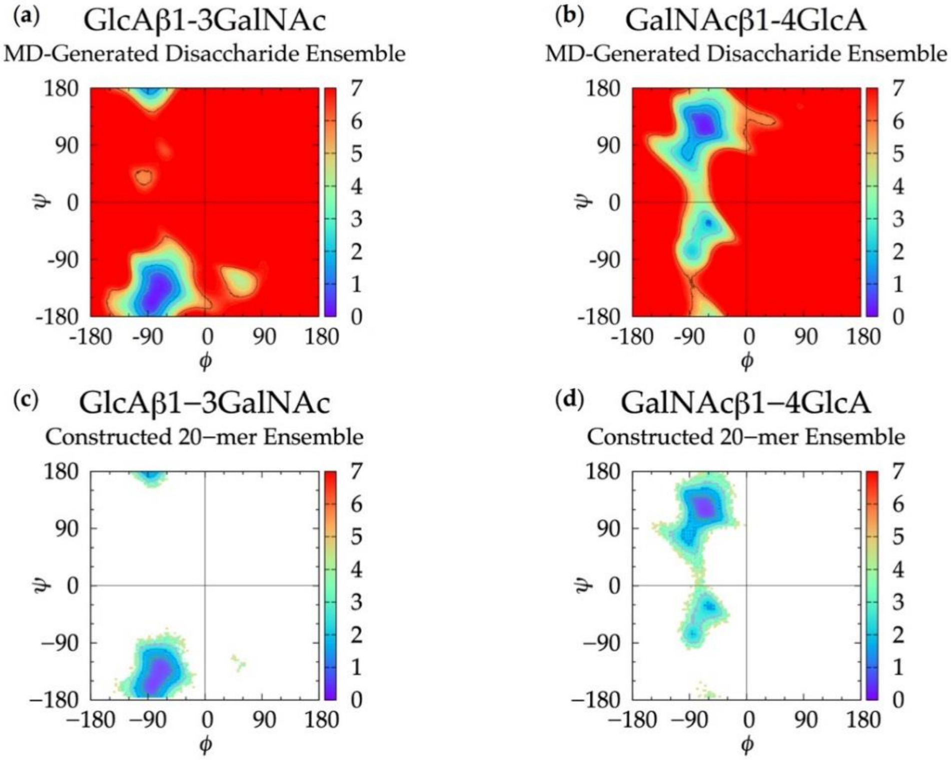

Figure S9. $\Delta G(\phi,\psi)$ plots for GlcAβ1-3GalNAc and GalNAcβ1-4GlcA glycosidic linkages in (**a**,**b**) non-sulfated chondroitin disaccharides simulated using MD with ABF on glycosidic linkage dihedrals (ϕ, ψ separated into 1° bins) and (**c**,**d**) 20-mer ensembles constructed using glycosidic linkage dihedral probabilities from ABF MD-generated disaccharides and standard force-field monosaccharide ring geometries (ϕ, ψ separated into 2.5° bins); contour lines every 1 kcal/mol; in ABF MD simulations, all values of ϕ, ψ were sampled but contours for data with $\Delta G(\phi, \psi) > 7$ kcal/mol are given values of 7 kcal/mol in plots (a,b) for clarity (shown in red).

(**a**)

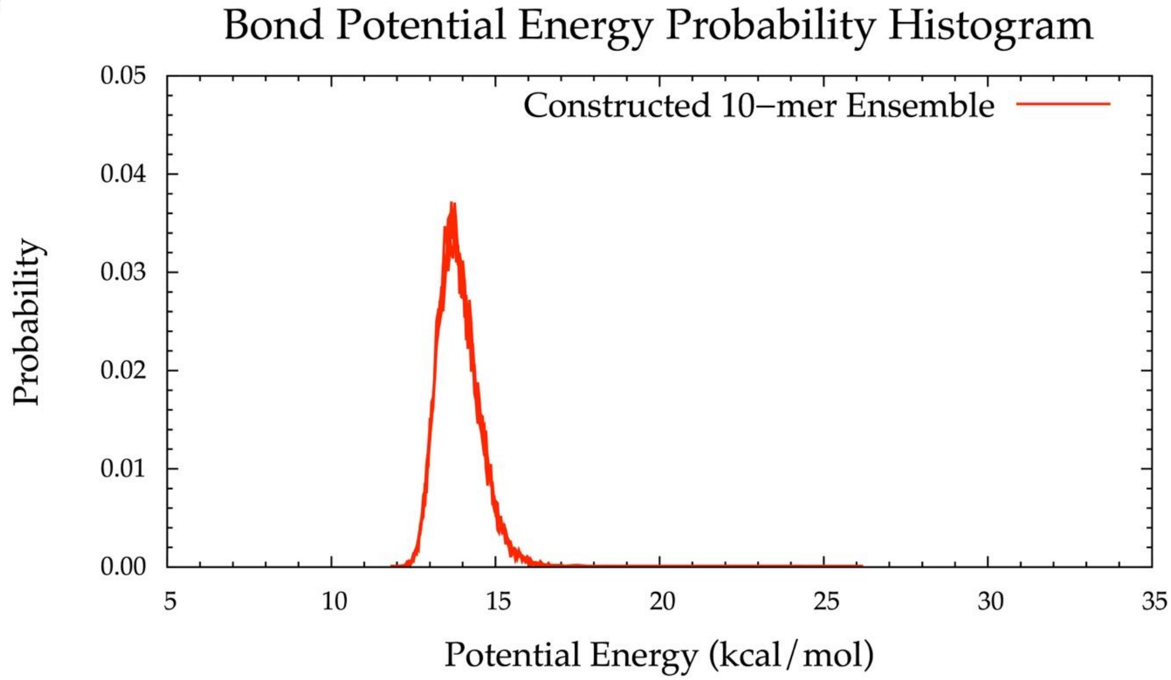

Bond Potential Energy Probability Histogram
Constructed 10−mer Ensemble
Probability
0.05
0.04
0.03
0.02
0.01
0.00
5
10
15
20
25
30
35
Potential Energy (kcal/mol)

(**b**)

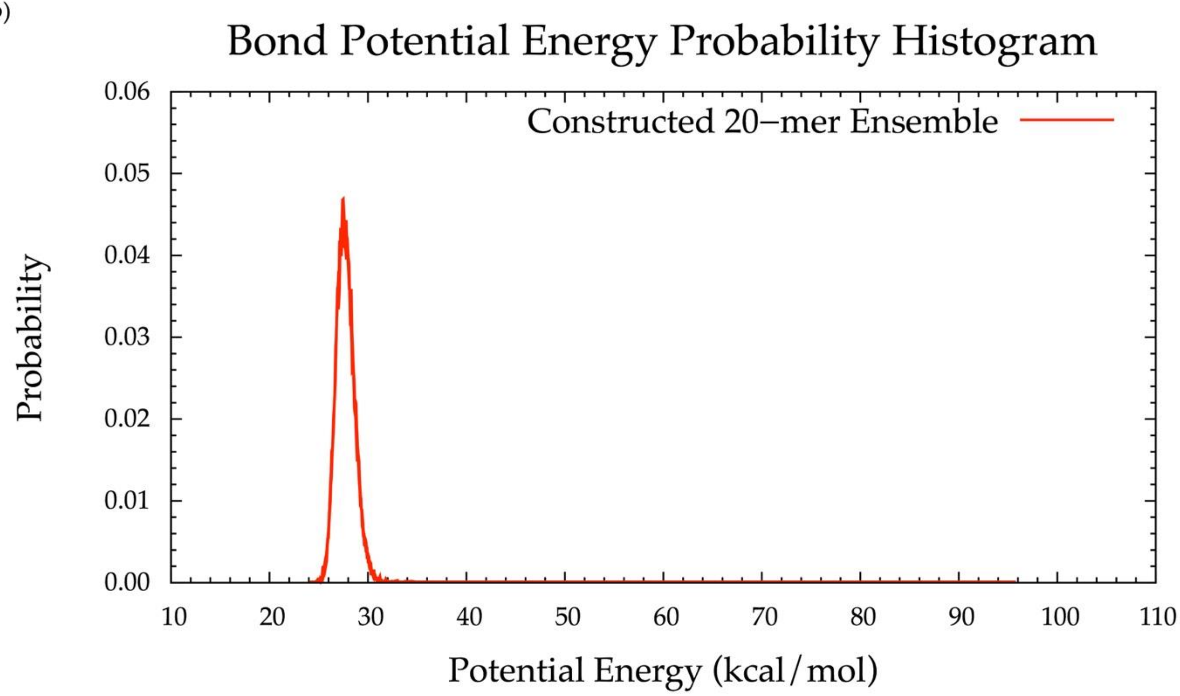

Bond Potential Energy Probability Histogram
Constructed 20−mer Ensemble
Probability
0.06
0.05
0.04
0.03
0.02
0.01
0.00
10
20
30
40
50
60
70
80
90
100
110
Potential Energy (kcal/mol)

(**c**)

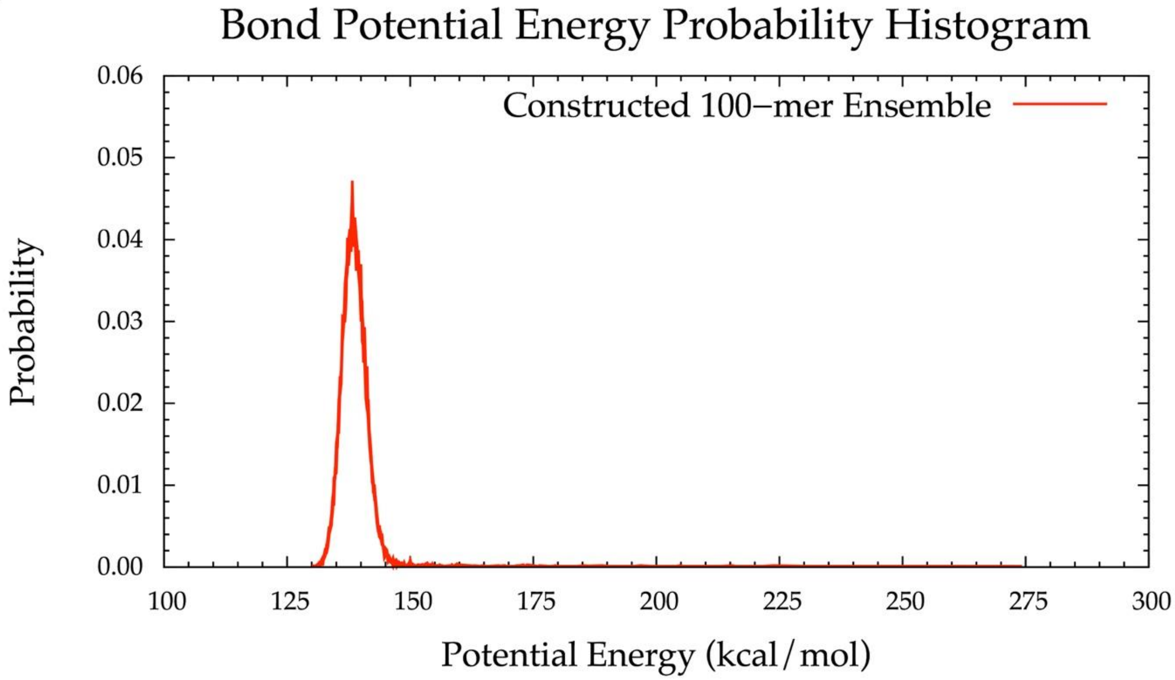

(**d**)

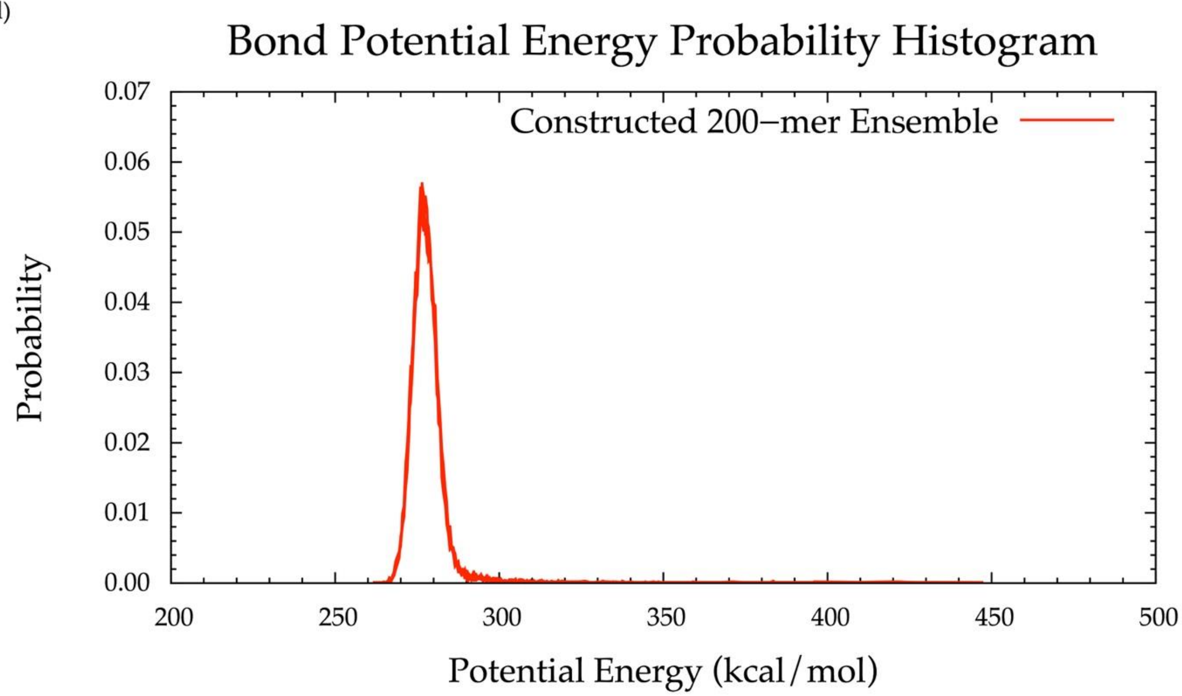

Figure S10. Bond energy distribution probability histograms from constructed ensembles of the (**a**) 10-mer (cutoff = 115.49 kcal/mol), (**b**) 20-mer (cutoff = 128.85 kcal/mol), (**c**) 100-mer (cutoff = 274.89 kcal/mol), and (**d**) 200-mer (cutoff = 449.26 kcal/mol).

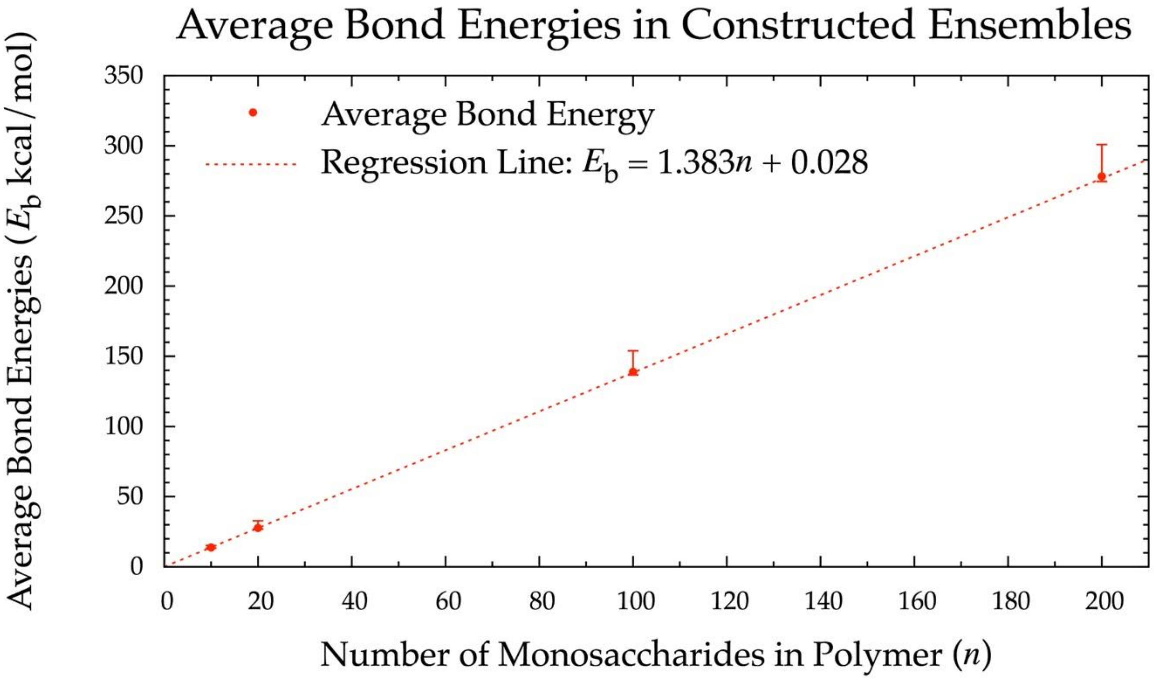

Figure S11. Average bond energies, standard deviations (calculated by fitting energies to gaussian curve), and regression line as a function of polymer length. Of note, (1) the regression equation calculated using only 10-mer and 20-mer constructed data: $E_b = 1.383n + 0.028$ (regression line plotted) closely matches (2) the regression equation calculated using 10-, 20-, 100-, and 200-mer constructed data: $E_b = 1.391n - 0.139$. Average bond energies of the 100-mer and 200-mer predicted by regression equation (1), $E_{b,predicted}(100) = 138.3$ kcal/mol and $E_{b,predicted}(200) = 276.6$ kcal/mol, are within 2 kcal/mol of the true averages, $E_b(100) = 138.8$ kcal/mol and $E_b(200) = 278.2$ kcal/mol.

References

1. Faller, C.E.; Guvench, O. Sulfation and cation effects on the conformational properties of the glycan backbone of chondroitin sulfate disaccharides. *J. Phys. Chem. B.* **2015**, *119*, 6063-6073, doi:10.1021/jp511431q.
2. Sattelle, B.M.; Shakeri, J.; Roberts, I.S.; Almond, A. A 3D-structural model of unsulfated chondroitin from high-field NMR: 4-sulfation has little effect on backbone conformation. *Carbohydr. Res.* **2010**, *345*, 291-302.
3. Darve, E.; Rodríguez-Gómez, D.; Pohorille, A. Adaptive biasing force method for scalar and vector free energy calculations. *J. Chem. Phys.* **2008**, *128*, 144120.
4. Henin, J.; Fiorin, G.; Chipot, C.; Klein, M.L. Exploring multidimensional free energy landscapes using time-dependent biases on collective variables. *J. Chem. Theory Comput.* **2009**, *6*, 35-47.
